# Age-Related Risk After Kidney Transplantation: A Comprehensive Analysis of Infection Burden, Graft Outcomes, and Mortality

**DOI:** 10.3389/ti.2025.15267

**Published:** 2026-01-07

**Authors:** Iris Schröter, Daniela Schindler, Martin Zeier, Thomas Giese, Claudia Sommerer

**Affiliations:** 1 Department of Nephrology, University Hospital Heidelberg, Heidelberg, Germany; 2 German Centre for Infection Research (DZIF), Munich, Germany; 3 Department of Immunology, University Hospital Heidelberg, Heidelberg, Germany; 4 German Centre for Infection Research (DZIF), Heidelberg, Germany

**Keywords:** kidney transplantation, elderly recipients, age, infections, fungal

## Abstract

Given the increasing number of kidney transplantation in elderly recipients, understanding age-specific risks is essential for optimized post-transplant care. We analyzed 572 kidney transplant recipients from the DZIF Transplant Cohort (2012–2023), stratified by age: <40 (n = 146), 40–60 (n = 279), >60 years (n = 147). Outcomes included infection burden, graft outcomes, and mortality over a median follow-up of 5 years. Multivariable Cox models with inverse probability weighting, adjusted for clinical confounders, was applied. In older recipients, the unadjusted 5-year rates of graft failure, mortality, and infections were significantly higher—both overall and for specific types, including pneumonia, urinary tract infections, invasive opportunistic infections, and multidrug-resistant infections. After adjustment, age remained only independently associated with mortality (HR = 6.21, p = 0.02), but not with overall infection burden or graft loss. Older patients exhibited a shift in pathogen prevalence, particularly for *Pseudomonas aeruginosa* and more severe herpesvirus infections, as well as higher infection-related morbidity, which contributed to graft failure. The first post-transplant year was critical, with infection burden strongly predicting graft failure (HR 1.16, p < 0.01). Age alone generally does not predict adverse transplant outcomes. Post-transplant care in elderly recipients should focus on early infection control with pathogen-targeted surveillance.

## Graphical Abstract

**Figure:**
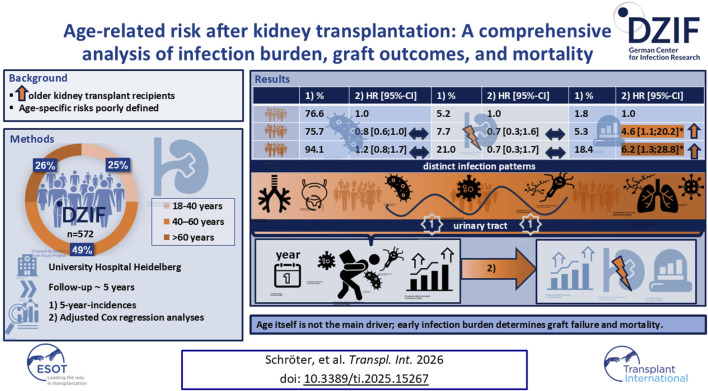

## Introduction

The prevalence of end-stage renal disease (ESRD) continues to rise in the aging population, making kidney transplantation increasingly common among older recipients [1–3]. This trend reflects demographic shifts and the increasing recognition of transplantation as a viable treatment option for selected elderly patients [1, 2, 4, 5]. However, older recipients face particular challenges due to immunosenescence, which alters immune system function and increases sensitivity to immunosuppressive therapy [6, 7]. This, together with greater frailty and a higher burden of comorbidities, complicates clinical management [7].

Guidelines emphasize emphasize individualized assessment rather than age-based exclusion [2]. Nevertheless, the protocols for immunosuppression, prophylaxis, and post-transplant monitoring are largely consistent across all age groups, due to limited data on age-specific complications [8, 9]. Older age is generally considered a universal risk factor for higher infection rates, early hospital readmissions, reduced graft function, increased graft loss, and mortality [7, 10–22]. The increasing number of older transplant recipients underscores the urgent need for a more nuanced understanding of age-related risks. However, there are a few studies that comprehensively evaluate these outcomes in parallel and adequately account for confounding variables. Furthermore, long-term data from Western European cohorts remain scarce, particularly on the type, severity, and timing of infections in older recipients. Most studies are limited by small sample sizes and focus primarily on infections occurring within the first year [11, 17, 18, 23]. This study investigates the association between recipient age and various short- and long-term post-transplant outcomes, including infection burden, graft function, acute rejection, graft loss, and mortality. The primary objective is to assess whether age independently predicts these outcomes after adjusting for relevant confounders. Secondary objectives are to characterize infection types, their timing, severity, and pathogens in different age groups. We hypothesize that although older age is initially associated with worse outcomes, this association will largely persist after extensive adjustment for selected endpoints. Furthermore, we expect to identify age-related differences in infection profiles, potentially leading to more targeted prevention and surveillance strategies.

## Patients and Methods

### Study Cohort, Ethics, and Follow-Up

The DZIF Transplant Cohort is a multicenter, prospective study within the framework of the German Center for Infection Research (DZIF) that focuses on transplant recipients and their infection risks [24]. This study specifically includes adult patients who received a kidney or simultaneous pancreas-kidney transplant at the University Hospital Heidelberg between January 2012 and December 2023. The Ethics Committee of the Medical Faculty of Heidelberg University approved for the study (No. S-585/2013), and all participants provided written informed consent. Clinical events were systematically recorded and evaluated, including all events up to December 2024. Follow-up examinations were performed at baseline, 3, 6, 9, and 12 months after transplantation, with additional annual visits thereafter or as clinically needed in case of complications. Clinical, laboratory, and demographic data were collected from medical records [24]. All recipients were followed for at least 1 year post-transplantation, unless death or graft loss occurred earlier.

### Immunosuppressive Regimen

The standard immunosuppressive protocol consisted of a calcineurin inhibitor - either tacrolimus (Tac) or ciclosporin A (CsA) - in combination with mycophenolate sodium (1.44 g/day) or mycophenolate mofetil (2 g/day), and methylprednisolone. Target trough (C_0_) levels for Tac were: month 1 (6–9 ng/mL), month 3 (5–8 ng/mL), thereafter (4–7 ng/mL); and for CsA: month 1 (150–180 ng/mL), month 3 (100–150 ng/mL), thereafter (80–120 ng/mL). Induction therapy included either basiliximab or thymoglobulin. Patients with previous transplants or high levels of donor-specific anti-HLA antibodies (DSA) were classified as highly sensitized. Additional desensitization strategies such as plasmapheresis or immunoadsorption were used in these patients, as well as in AB0-incompatible recipients.

### Prophylaxis and Surveillance Strategy

Prophylaxis and monitoring were performed according to the KDIGO 2009 guidelines [25]. For Pneumocystis jirovecii prophylaxis, trimethoprim-sulfamethoxazole (800 mg/160 mg) was routinely administered three times a week for the first 6 months after transplantation. Standard antiviral prophylaxis with valganciclovir was administered to all Cytomegalovirus (CMV) immunoglobulin G (IgG)–positive recipients and recipients of CMV IgG–positive donor organs for at least 3 months. In high-risk (D+/R–) cases, 6 months of antiviral prophylaxis was recommended. The dosage was adjusted according to renal function. *Candida* prophylaxis with oral nystatin was provided within the first one to 3 months if more than 20 mg of methylprednisolone was administered daily.

### Definitions of Infectious Complications

All infections requiring hospitalization were included. Diagnoses were made by the treating physician. Data collected included clinical presentation, laboratory and microbiological/virological findings, diagnostic procedures, treatment, disease course, and infection-related outcomes. Detailed infection definitions are provided in the (Supplementary Table S1).

### Acute Rejections

Acute rejection was diagnosed by histopathological examination of renal biopsies by a trained local kidney pathologists based on morphological assessment and immunohistochemical markers. Rejection episodes were classified according to the Banff criteria [26].

### Statistical Methods

Baseline characteristics were summarized using means ± standard deviations or medians with interquartile ranges for continuous variables and number with percentages for categorical variables. Group comparisons were performed using Pearson’s chi-squared test or Fisher’s exact test for categorical data, and Student’s t-test or Mann–Whitney U test for continuous variables, as appropriate. Survival probabilities were estimated using the Kaplan–Meier method and compared using the log-rank test. The association between recipient age (<40, 40–60, >60 years) was first explored through univariable Cox proportional hazards regression models. To estimate independent effects, propensity scores were derived from a multinomial logistic regression model including key baseline covariates. Inverse probability weighting (IPW) based on these propensity scores was then applied to create a weighted pseudo-population minimizing residual confounding. Subsequently, IPW-weighted multivariable Cox regressions were fitted to assess independent associations between recipient age and outcomes. Variables with a p-value <0.1 in the weighted univariate analyses and clinically relevant variables were entered into multivariable models, while age group was retained in all models. Hazard ratios (HRs) with 95% confidence intervals (CIs) were reported; *p* < 0.05 was considered statistically significant.

Sensitivity analyses included unweighted and trimmed (2.5th–97.5th percentile) IPW models, as well as models with and without adjustment for first-year infection burden (Supplementary Table S3). To account for potential COVID-19 bias, follow-up was additionally censored at 1 March 2020, or modeled with a pandemic indicator (March 2020–December 2022).

Death before graft failure was treated as a censoring event. Variables were handled by complete-case analysis within each model. Given the overall low proportion of missing data (<5% across key variables), multiple imputation was not deemed necessary.

Risk factors for infection burden within the first post-transplant year were analyzed using negative binomial regression.

All statistical analyses were conducted using R software version 2024.12.0 + 467.

## Results

A total of 572 adult kidney transplant recipients (62.6% male, mean age 49 ± 14 years) were included and divided into three age groups: <40 years (25.5%), 40–60 years (48.8%), and >60 years (25.7%). Clinical and demographic characteristics are summarized in Table 1. The median follow-up was 61 months (IQR 30–87), with no significant differences between groups.

**TABLE 1:** Clinical and demographic characteristics of the total cohort and stratified by age group.

	Total	<40 Years	40–60 Years	>60 Years
No. of patients	572	147	279	146
Demographics				
Age (mean ± SD)	49.4 ± 13.5	30.8 ± 5.5	52.2 ± 5.5	65.6 ± 3.4
Male gender (%)	62.6	59.9	62.4	65.8
Clinical data				
Body mass index (mean ± SD) in kg/m^2^	26.5 ± 4.7	25.0 ± 4.6	27.0 ± 4.9	27.0 ± 4.1
Diabetes mellitus (%)	20.6	9.6	15.8	25.3
Cause of ESRD				
Glomerulonephritis (%)	26.9	17.7	30.5	29.5
ADPKD (%)	17.5	10.2	18.3	23.3
Diabetes mellitus (%)	8.2	5.4	9.3	8.9
Nephrosclerosis (%)	10.5	6.8	10.0	14.4
Intestinal nephritis (%)	3.0	2.7	3.2	2.7
Vasculitis and collagenoses (%)	9.3	12.9	9.4	5.5
Urological diseases (%)	4.7	10.2	3.2	2.1
Other hereditary diseases (%)	4.9	15.0	2.2	1.4
Other (%)	15.0	19.0	14.0	12.3
Donor characteristics				
Donor age	55.4 ± 14.9	52.2 ± 11.1	52.2 ± 14.6	64.7 ± 15.0
Male donor	43.3	44.5	44.1	40.4
CMV IgG serology				
D+/R- (%)	20.2	19.0	20.6	20.5
D+/R+ (%)	33.2	34.0	30.0	38.4
D-/R+ (%)	24.6	23.1	23.8	27.4
D-/R- (%)	22.1	23.8	25.6	13.7
Type of transplantation and immunology				
Living donation (%)	33.6	53.7	33.7	13.0
AB0i (%)	4.6	6.8	4.3	2.7
High sensitization (%)	9.3	10.2	11.8	3.4
ESP (%)	12.2	0.0	0.0	
Pancreas-kidney (%)	3.3	4.1	4.7	0.0
Cold ischemia time (min), mean ± SD	565.5 ± 378.5	448.5 ± 392.1	587.2 ± 397.8	641.0 ± 292.7
Mean number of HLA matches (±SD)	2.9 ± 1.6	3.1 ± 1.4	3.0 ± 1.6	2.4 ± 1.6
Mean number of HLA mismatches (±SD)	2.8 ± 1.6	2.7 ± 1.3	2.7 ± 1.7	3.3 ± 1.8
Immunosuppression				
Induction therapy				
Basiliximab (%)	75.0	73.5	72.0	82.1
Thymoglobuline (%)	25.0	26.5	28.0	17.9
Rituximab (%)	9.4	14.3	10.0	3.4
Plasmapheresis (%)	20.1	26.5	21.9	10.3
Immunadsorption (%)	3.8	5.4	3.9	2.1
Maintenance therapy at discharge				
Tacrolimus + MPA/MMF + Steroids (%)	75.2	80.3	76.3	67.8
Ciclosporine A + MPA/MMF + Steroids (%)	24.0	19.0	23.7	29.5
Antimicrobial prophylaxis				
Valganciclovir (%)	62.9	65.3	59.9	65.8
Cotrimoxazol (%)	84.1	88.4	83.5	80.8
Dapson (%)	15.9	11.6	16.5	19.2
Post-operative course				
Delayed graft function [1]	27.0	22.4	24.5	36.3

Missing values were excluded. Variables were handled by complete case analysis. Abbreviations: AB0i = AB0 incompatibility, ADPKD = autosomal dominant polycystic kidney disease, C0 = trough level, CMV = cytomegalovirus, D+/− = CMV IgG donor positive/negative, ESRD = end-stage renal disease, ESP = Eurotransplant Senior Program, HLA = human leukocyte antigen, IgG = immunoglobulin G, IQR = interquartile range, Md = median, MMF = mycophenolate mofetil, MPA = mycophenolic acid, R+/− = CMV IgG recipient positive/negative, SD = standard deviation [1]. Defined as the requirement for dialysis within the first 7 days after kidney transplantation, excluding dialysis performed solely for hyperkalemia.

**FIGURE 1:**
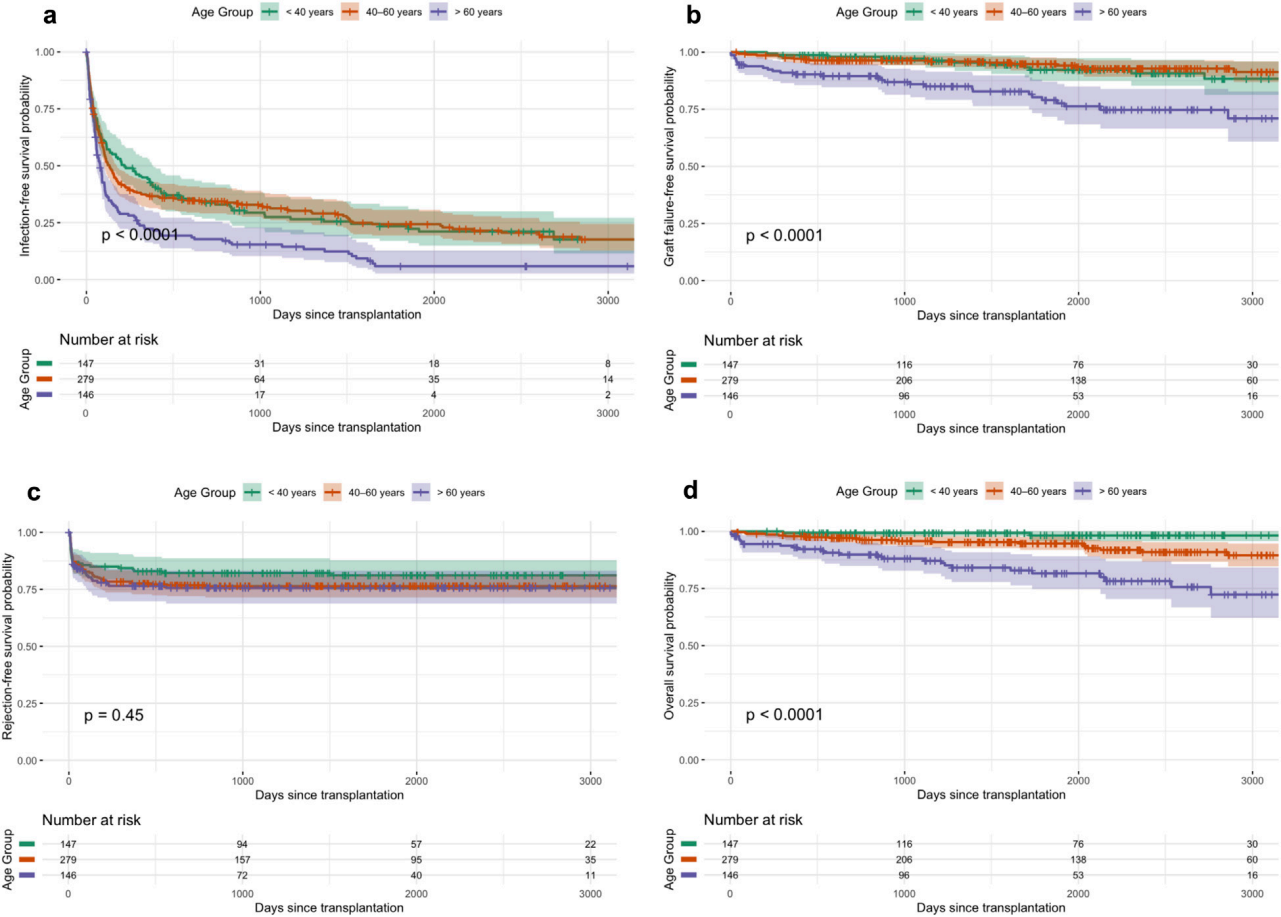
**(a–d)** Kaplan-Meier curves for long-term infection-free **(a)**, graft-failure-free **(b)**, rejection-free survival **(c)**, and overall survival **(d)** across age groups.

Figures 1a–d displays Kaplan–Meier curves for long-term infection-free (a), graft-failure-free (b), rejection-free (c), and overall survival (d), stratified by age.

### GFR Dynamics and Immunosuppression

The incidence of delayed graft function (DGF) increased significantly with recipient age, rising from 22.4% in recipients <40 years to 36.3% in those >60 years (p = 0.012, Table 1). Across all age groups, there was a significant improvement in estimated glomerular filtration rate (eGFR) from month 1 to month 3, with the greatest relative increase observed in recipients >60 years (median +50.2% from the baseline median of 24.5 mL/min/1.73 m^2^). Younger recipients maintained higher absolute eGFR levels throughout the first year (Figure 3a). In recipients >60 years, lower tacrolimus doses were required to achieve comparable and adequate drug levels (Figure 3b).

### Overall Infection Incidences and Age-Related Patterns of Bacterial, Viral, and Fungal Infections

Short and long-term infection incidences are provided in Table 2. Older recipients had the highest overall infection rates, with a 5-year incidence of 94.1% (p < 0.001; Table 2). Bacterial infections were more frequent in older patients (>60: 77.0%, p < 0.0001), while viral infections occurred at similar rates across all age groups (p = 0.761). Fungal infections were significantly more common in older recipients (>60: 9.8% vs. <40: 0.8%, 40–60: 3.3%; p = 0.002).

**TABLE 2:** Age-stratified short- and long-term incidences of clinical outcomes.

Outcome and time interval	Total	<40 Years	40–60 Years	>60 Years	p
Mortality					
Overall day 0–30 day 0–180 day 0–365 year 2 year 3 year 5	17.1 [11.5; 25.5] 0.5 [0.3; 1.6] 1.9 [1.1; 3.5] 3.0 [1.9; 4.8] 4.2 [2.8; 6.2] 5.2 [3.6; 7.5] 7.6 [5.5; 10.4]	1.8 [0.4; 7.5] 0.0 [0.0; 0.0] 0.0 [0.0; 0.0] 0.7 [0.1; 0.5] 0.7 [0.1; 0.5] 0.7 [0.1; 0.5] 1.8 [0.4; 7.5]	12.3 [7.5; 20.1] 0.0 [0.0; 0.0] 1.1 [0.4; 3.3] 2.2 [1.0; 3.8] 2.9 [1.5; 5.8] 4.3 [2.4; 7.6] 5.3 [3.1; 9.1]	51.8 [28.8; 93.1] 2.1 [0.7; 6.3] 5.6 [2.8; 10.9] 7.0 [3.9; 12.8] 10.2 [6.2; 16.7] 11.9 [7.5; 18.9] 18.4 [12.4; 27.2]	<0.0001
Graft failure					
Overall day 0–30 day 0–180 day 0–365 year 2 year 3 year 5	14.4 [10.9; 19.0] 0.9 [0.4; 2.1] 2.3 [1.3; 3.9] 4.2 [2.9; 6.3] 5.0 [3.5; 7.1] 6.1 [4.3; 8.4] 9.8 [7.4; 12.9]	8.7 [5.2; 14.6] 0.0 [0.0; 0.0] 1.4 [0.5; 3.8] 2.9 [1.5; 5.7] 3.6 [2.0; 6.7] 3.6 [2.0; 6.7] 5.2 [3.0; 8.9]	11.7 [6.3; 21.8] 0.0 [0.0; 0.0] 0.0 [0.0; 0.0] 1.4 [0.3; 5.4] 2.1 [0.7; 6.4] 2.9 [1.1; 7.6] 7.7 [4.1; 14.6]	29.0 [20.0; 42.2] 3.4 [1.4; 8.1] 6.2 [3.3; 11.6] 9.7 [5.0; 16.0] 10.5 [6.5; 16.9] 14.0 [9.2; 21.4] 21.0 [14.6; 30.2]	<0.0001
Acute rejection					
Overall day 0–30 day 0–180 day 0–365 year 2 year 3 year 5	22.6 [19.3; 26.3] 13.9 [11.3; 17.0] 19.2 [16.3; 22.8] 20.9 [17.8; 24.5] 22.0 [18.9; 25.7] 22.3 [19.1; 26.0] 22.6 [19.3; 26.3]	18.9 [13.4; 26.6] 12.9 [8.5; 19.7] 15.0 [10.2; 22.0] 15.7 [10.8; 22.8] 17.8 [12.5; 25.2] 17.8 [12.5; 25.2] 18.9 [13.4; 26.6]	23.6 [19.1; 29.2] 14.0 [10.4; 18.7] 20.2 [16.0; 25.5] 22.4 [18.0; 27.9] 23.2 [18.7; 28.7] 23.6 [19.1; 29.2] 23.6 [19.1; 29.2]	24.3 [18.1; 32.7] 14.6 [9.8; 21.7] 22.0 [16.1; 30.0] 23.5 [17.4; 31.7] 24.3 [18.1; 32.7] 24.3 [18.1; 32.7] 24.3 [18.1; 32.7]	0.4480
Infection (any)					
Overall day 0–30 day 0–180 day 0–365 year 2 year 3 year 5	85.4 [81.8; 89.1] 21.7 [18.6; 25.4] 57.4 [53.4; 61.6] 65.3 [61.5; 69.4] 69.9 [66.2; 73.9] 73.7 [70.0; 77.5] 80.6 [77.0; 84.3]	82.4 [75.1; 90.3] 21.8 [16.0; 29,6] 46.3 [38.9; 55.1] 57.2 [49.8; 65.8] 66.2 [58.8; 74.5] 72.5 [65.3; 80.5] 76.6 [69.5; 84.4]	82.3 [76.8; 88.3] 19.7 [15.6; 25.0] 57.0 [51.5; 63.2] 63.3 [57.9; 69.3] 65.7 [60.3; 71.6] 68.7 [63.3; 74.6] 75.7 [70.3; 81.5]	94.1 [89.7; 98.8] 25.6 [19.4; 33.8] 69.6 [62.4; 77.7] 77.7 [71.1; 84.9] 82.2 [76.1; 88.9] 84.6 [787; 90.9] 94.1 [89.7; 98.8]	<0.0001
Bacterial infection					
Overall day 0–30 day 0–180 day 0–365 year 2 year 3 year 5	64.5 [59.6; 69.9] 17.5 [14.7; 20.9] 35.3 [31.6; 39.5] 40.8 [36.9; 45.0] 44.9 [41.0; 49.3] 49.7 [45.6; 54.1] 56.7 [52.5; 61.4]	62.8 [53.7; 73.5] 17.0 [11.9; 24.3] 25.2 [19.0; 33.3] 32.0 [25.3; 40.6] 37.1 [30.0; 45.9] 42.9 [35.4; 52.0] 54.0 [45.8; 63.6]	55.6 [48.6; 63.7] 15.4 [11.7; 20.3] 32.2 [27.1; 38.2] 35.5 [30.3; 41.6] 39.0 [33.6; 45.2] 42.4 [36.8; 48.8] 47.9 [41.9; 54.6]	85.2 [75.1; 96.6] 22.1 [16.3; 30.0] 52.0 [44.4; 61.0] 59.5 [51.8; 68.2] 64.8 [57.3; 73.3] 71.1 [63.7; 79.4] 77.0 [69.6; 85.1]	<0.0001
Viral infection					
Overall day 0–30 day 0–180 day 0–365 year 2 year 3 year 5	64.8 [52.7; 79.8] 2.3 [1.3; 3.9] 28.4 [24.9; 32.9] 36.2 [32.4; 40.4] 41.4 [37.4; 45.7] 45.1 [41.0; 49.5] 49.5 [45.3; 54.1]	76.0 [48.9;-] 2.7 [1.0; 7.2] 25.9 [19.7; 34.0] 35.5 [28.5; 44.1] 42.1 [34.7; 51.0] 46.4 [38.8; 55.6] 47.5 [39.8; 56.7]	57.4 [49.9; 65.9] 2.2 [1.0; 4.7] 29.0 [24.1; 34.9] 37.1 [31.8; 43.2] 40.5 [35.1; 46.8] 43.8 [38.2; 50.2] 48.7 [42.8; 55.4]	67.3 [53.9; 84.1] 2.1 [0.7; 6.4] 30.6 [23.8; 39.4] 35.2 [28.0; 44.2] 42.6 [34.9; 51.9] 46.3 [38.4; 55.9] 53.6 [45.1; 63.7]	0.761
Fungal infection					
Overall day 0–30 day 0–180 day 0–365 year 2 year 3 year 5	4.6 [3.0; 6.9] 0.7 [0.3; 1.9] 1.9 [1.1:3.5] 2.5 [1.5; 4.2] 3.3 [2.1; 5.1] 3.9 [2.6; 6.0] 4.2 [2.8; 6.3]	0.8 [0.1; 5.4] - - - 0.8 [0.1; 5.4] 0.8 [0.1; 5.4] 0.8 [0.1; 5.4]	4.0 [2.1; 7.6] 0.4 [0.1; 2.5] 2.2 [1.0; 4.8] 2.5 [1.2; 5.3] 3.3 [1.7; 6.2] 3.3 [1.7; 6.2] 3.3 [1.7; 6.2]	9.8 [5.7; 16.9] 2.1 [0.7; 6.4] 3.5 [1.5; 8.3] 5.0 [2.4; 10.4] 5.8 [3.0; 11.4] 8.7 [4.9; 15.5] 9.8 [5.7; 16.9]	0.002
Urinary tract infection					
Overall	29.8 [26.2; 33.8]	22.5 [16.7; 30.4]	28.4 [23.5; 34.2]	40.4 [33.0; 49.5]	0.003
Pneumonia					
Overall	23.9 [19.4; 29.6]	20.0 [12.3; 32.6]	18.1 [13.3; 24.7]	45.1 [30.6; 66.5]	<0.0001
Upper respiratory tract infection					
Overall	23.6 [19,0.1; 29.3]	25.5 [18.1; 35.8]	24.1 [18.0; 32.4]	22.2 [12.2; 40.4]	0.167
Gastrointestinal infection					
Overall	27.9 [17.4; 44.5]	25.1 [16.8; 37.5]	24.2 [10.4; 56.2]	35.1 [23.1; 53.4]	<0.0001
Sepsis					
Overall	21.8 [17.3; 27.5]	22.7 [14.1; 36.6]	20.1 [14.3; 28.1]	23.5 [15.9; 34.7]	0.236
Invasive opportunistic infection					
Overall	9.1 [6.3; 13.1]	3.4 [1.3; 9.0]	10.0 [5.9; 16.9]	12.7 [7.8; 20.6]	0.011

Cumulative-incidences with 95%-confidence-intervals.

### Urinary Tract Infections

**FIGURE 2:**
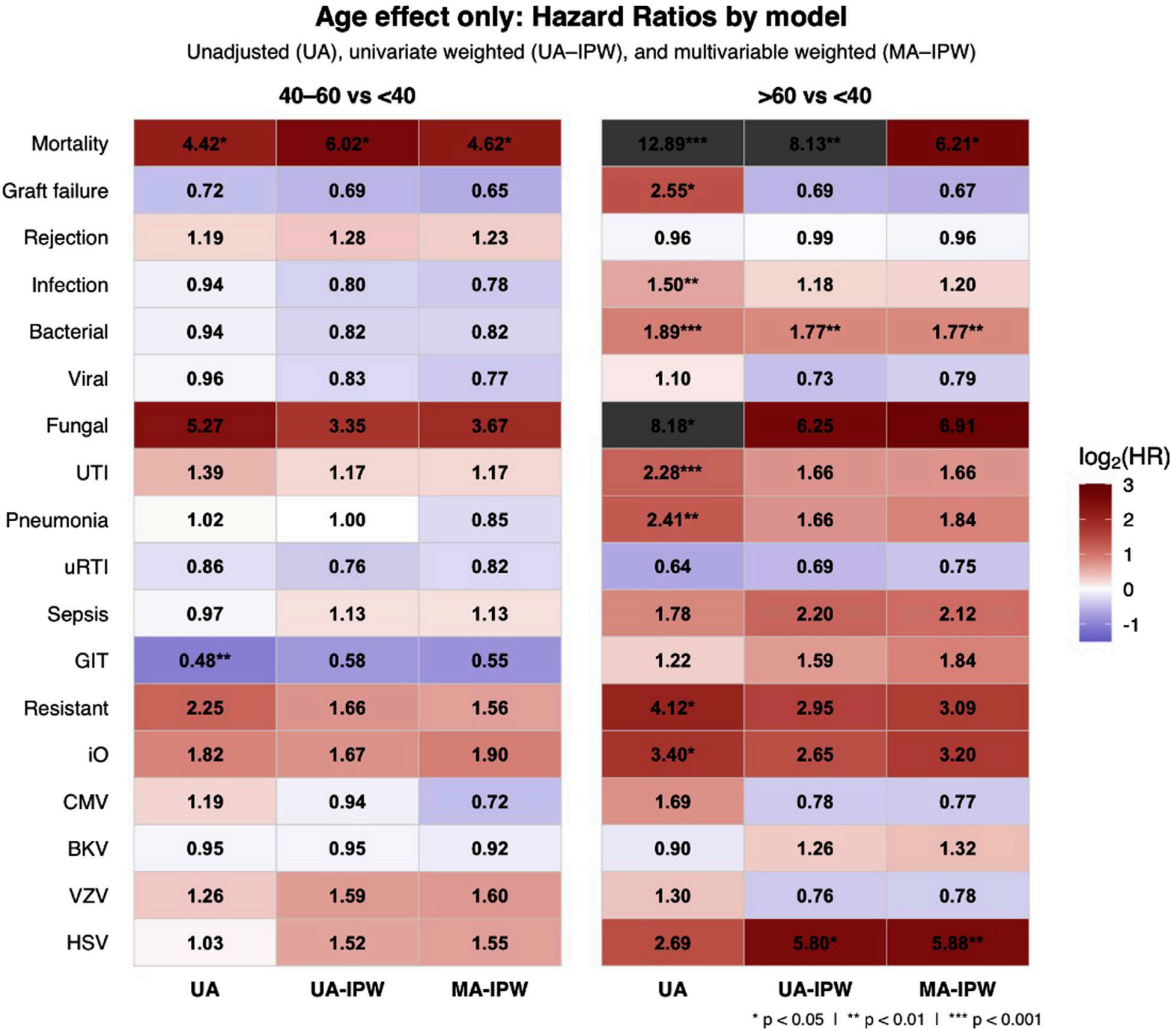
Heatmap of log_2_-transformed hazard ratios (HRs) showing the independent effect of age on clinical outcomes by age group using a three-step IPW-adjusted modeling approach. The heatmap displays HRs with 95% confidence intervals (CIs) comparing age groups 40–60 and >60 years to the reference group <40 years. Colors indicate effect size. The three-step approach includes unadjusted Cox (UA), inverse probability weighted univariate (UA-IPW), and IPW-adjusted multivariable (MA-IPW) models, where age group and variables with p < 0.1 in UA-IPW and clinically relevant variables were included in the MA-IPW model. All covariates analyzed and included are detailed in Table 2 and Supplementary Table S2. Abbreviations: UTI; urinary tract infection; uRTI; upper respiratory tract infection; GIT – gastrointestinal infection; iO – invasive opportunistic; CMV – cytomegalovirus; BKV – BK polyomavirus; VZV – varicella zoster virus; HSV – herpes simplex virus.

UTIs were the most common infection type across all age groups, accounting for approximately 34%–35% of all infections. Although older age was initially associated with a higher risk of UTI, this association was no longer significant after multivariable adjustment (Figure 2; Supplementary Table S2).

The number of UTIs per infected patient was highest in recipients <40 years (3.6 episodes), followed by >60 years (3.2) and 40–60 years (2.7). Across groups, *E. coli* (42%, 38%, 35%) and *Enterococcus* spp. (30%, 22%, 30%) were the leading uropathogens. *Pseudomonas aeruginosa* prevalence increased with age (6% in <40 vs. 18% in >60), as did *Enterobacter* spp. (2%, 4%, 5%), while *Klebsiella* spp. decreased (21%, 18%, 12%) (Supplementary Figure S2). Resistant uropathogens were more frequent in recipients >60 years (17.6% vs. 6.7% in <40), but age was not an independent risk factor for resistant infections after full adjustment (Figure 2; Supplementary Table S2).

Older recipients more often had multidrug-resistant Gram-negative infections causing urosepsis or transplant pyelonephritis (45.4%). In recipients <40 years, resistant infections were limited to uncomplicated lower UTIs. Patients with resistant infections averaged 7.7 ± 5.8 episodes during follow-up and 3.9 ± 3.2 in the first post-transplant year. Their 5-year mortality was 28.4% (95%-CI, 19.3–38.4). Graft failure occurred in 33.3%. Cumulative incidence rose with age (from 4.8% to 11.6%), but age was not an independent predictor after full adjustment (HR = 1.66, p = 022.39; Table 2; Figure 2).

### Distribution of Other Infection Types

**FIGURE 3:**
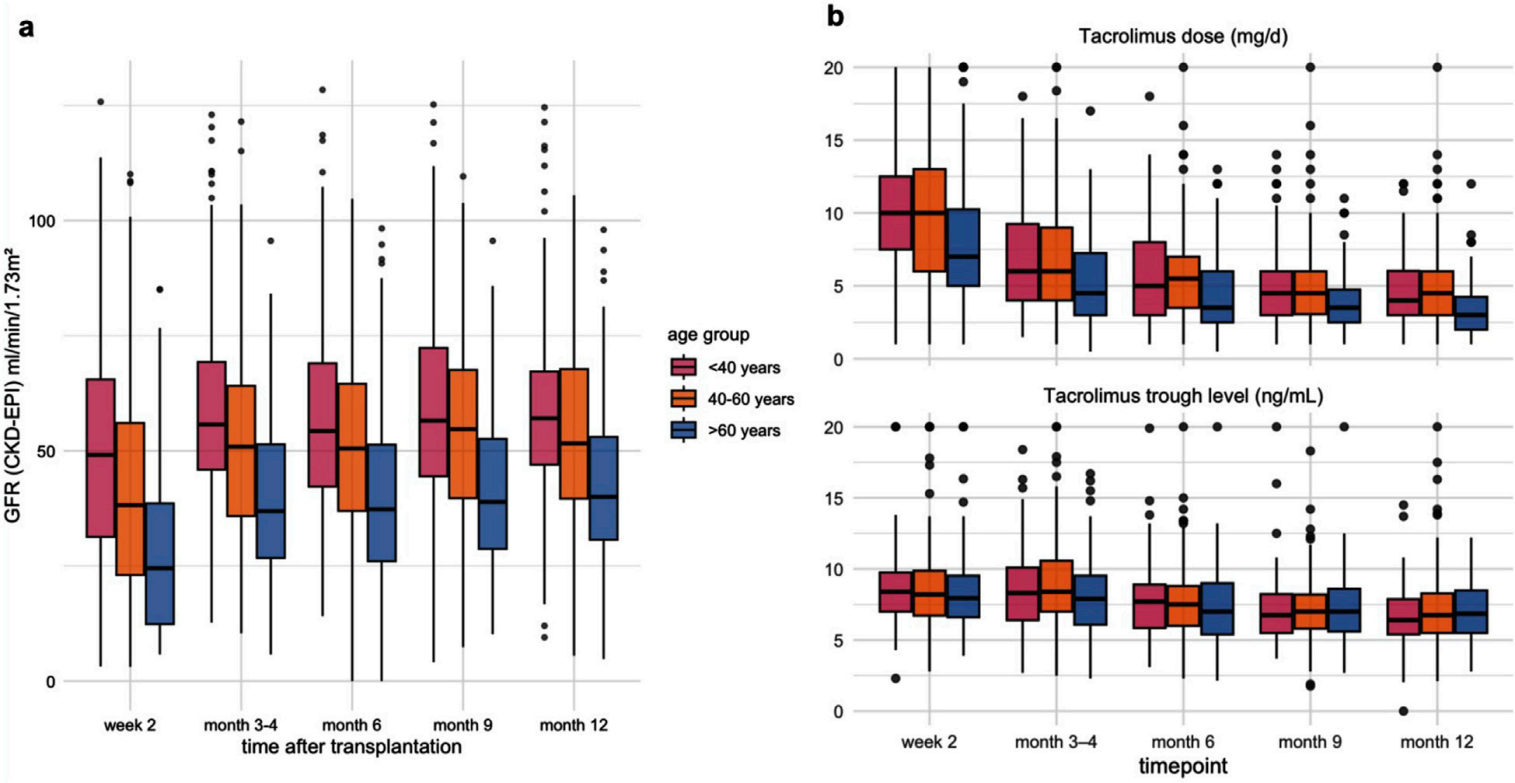
**(a)** Dynamics of estimated glomerular filtration rate (eGFR, CKD-EPI) during the first post-transplant year, stratified by recipient age group. Boxplots show median, interquartile range, and range (whiskers) of eGFR (mL/min/1.73 m^2^) at week 2, months 3–4, 6, 9, and 12 after kidney transplantation. **(b)** Tacrolimus dose and trough levels during the first post-transplant year by recipient age group. Boxplots display median values, interquartile ranges, and outliers at each time point (week 2, months 3–4, 6, 9, and 12).

Other types of infection showed age-related patterns. Pneumonia accounted for a higher proportion of infections with increasing age: 6.9% (<40 years) vs. 11.8% (>60 years) with a higher incidence in the elderly (Table 2), but this difference lost significance after adjustment (Supplementary Table S2). In contrast, upper respiratory tract infections decreased with age (10.3% vs. 3.2%). Gastrointestinal infections, pyelonephritis, and infections of unknown origin remained stable across all age groups (Table 3; Figure 3). Viral GI infections were more common in younger recipients, while *Clostridium difficile* was more prevalent in older groups. Sepsis rates remained stable across all age groups (8%–9%). The majority of sepsis cases (89.2%) originated from UTIs, mainly caused by Gram-negative bacteria (89.2%), particularly *E. coli* (52%) and *Klebsiella* spp. (20.4%). Sepsis due to pneumonia or soft tissue infections was rare, both accounted for 3.1% of all sepsis cases.

**TABLE 3:** Risk analyses for clinical outcomes using a three-step IPW-adjusted model.

Outcome and covariates	1) Univariate HR (95% CI)	p-value	2) Univariate IPW HR (95% CI)	p-value	3) Multivariate IPW HR (95% CI)	p-value
Mortality						
Agegroup 2	**4.14 [1.02;19.11]**	**0.0047**	**5.99 [1.06;8.25]**	**0.0191**	**4.62 [1.05;20.23]**	**0.0424**
Agegroup 3	**13.20 [3.09;56.36]**	**0.0005**	**8.07 [1.70;38.33]**	**0.0086**	**6.21 [1.34;28.81]**	**0.0198**
Male gender	1.37 [0.69; 2.69]	0.3663	1.80 [0.78; 4.14]	0.1671		
BMI (per 5 kg/m^2^)	**1.09 [1.03;1.15]**	**0.0032**	**1.95 [1.41;2.69]**	**<0.001**	**1.61 [1.11;2.33]**	**0.0112**
Diabetes mellitus	1.64 [0.83:3.21]	0.1536	**2.68 [1.07;6.72]**	**0.0361**	1.77 [0.68; 4.65]	0.2439
Donor age (per 10 years)	**1.07 [1.04;1.09]**	**<0.0001**	**1.79 [1.18;2.72**	**0.0061**	**1.13 [0.99;1.29]**	**0.0073**
Male donor	0.68 [0.37; 1.23]	0.2088	0.59 [0.26; 1.24]	0.1538		
Deceased donation	**4.59 [1.78;11.85]**	**0.0017**	**2.96 [1.06;8.25]**	**0.0380**	1.39 [0.32; 6.00]	0.6601
High sensitization	0.80 [0.25; 2.61]	0.8191	0.80 [0.24; 2.72]	0.7236		
Thymoglobuline	0.80 [0.35; 1.83]	0.5214	**0.51 [0.20;1.27]**	**0.1480**		
HLA mismatches (no.)	1.03 [0.84; 1.26]	0.7703	1.07 [0.84; 1.36]	0.5936		
AB0i	0.98 [0.58; 4.49]	0.3602	1.63 [0.38; 7.06]	0.5128		
Cold ischemia time (min)	**1.00 [1.00;1.00]**	**0.0038**	**1.00 [1.00;1.00]**	**0.0070**	1.00 [1.00; 1.00]	0.4204
Delayed graft function	**1.79 [0.94;3.40]**	**0.0146**	1.63 [0.76; 3.49]	0.2082		
CKD-EPI W2	**0.97 [0.95;0.98]**	**0.0002**	**0.73 [0.60;0.88]**	**0.0008**	0.88 [0.70; 1.10]	0.2629
CMV IgG D+	**1.57 [0.80;3.07]**	**0.1877**	1.02 [0.46; 2.27]	0.9582		
CMV IgG R+	**0.95 [0.50;1.78]**	**0.8619**	0.75 [0.35; 1.61]	0.4561		
CMV IgG D+/R-	**1.19 [0.56;2.50]**	**0.6493**	0.91 [0.36; 2.29]	0.8438		
No. of infections (y1)	**1.28 [1.15;1.44]**	**<0.0001**			**1.13 [1.05;1.29]**	**0.0307**
Graft failure						
Agegroup 2	0.71 [0.33; 1.55]	0.3892	0.70 [0.29; 1.67]	0.4188	0.65 [0.26; 1.62]	0.3537
Agegroup 3	**2.50 [1.22;5.14]**	**0.0124**	0.70 [0.27; 1.76]	0.4444	0.67 [0.26; 1.69]	0.3924
Male gender	0.93 [0.52; 1.65]	0.8051	0.88 [0.42; 1.84]	0.7332		
BMI (per 5 kg/m^2^)	1.01 [0.95; 1.07]	0.7424	1.12 [0.84; 1.49]	0.4498		
Diabetes mellitus	**1.94 [1.03;3.65]**	**0.0411**	1.67 [0.78; 3.57]	0.1876		
Donor age (per 10 years)	**1.06 [1.03;1.08]**	**<0.0001**	**1.69 [1.29;2.23]**	**0.0002**	**1.60 [1.21;2.12]**	**0.0009**
Male donor	1.53 [0.87; 2.68]	0.1381	**2.15 [1.04;4.42]**	**0.0381**	**2.21 [1.01;4.86]**	**0.0474**
Deceased donation	**4.12 [1.75;9.69]**	**0.0012**	**3.62 [1.32;9.90]**	**0.0121**		
High sensitization	1.81 [0.85; 3.85]	0.1260	2.11 [0.90; 4.95]	0.0848	2.63 [0.98; 7.07]	0.0560
Thymoglobuline	0.85 [0.41; 1.77]	0.6692	0.62 [0.26; 1.48]	0.2802		
HLA mismatches (no.)	1.10 [0.91; 1.32]	0.0509	1.18 [0.97; 1.44]	0.1008	1.12 [0.85; 1.46]	0.4244
AB0i	0.42 [0.06; 3.03]	0.3887	0.75 [0.11; 5.17]	0.7682		
Cold ischemia time (min)	**1.00 [1.00;1.00]**	**0.0096**	**1.00 [1.00;1.00]**	**0.0083**	1.00 [1.00; 1.00]	0.9494
Delayed graft function	**2.32 [1.32;4.08]**	**0.0034**	**2.67 [1.31;5.43]**	**0.0068**	1.15 [0.42; 3.18]	0.7822
CKD-EPI W2	**0.97 [0.96;0.99]**	**0.0001**	**0.76 [0.64;0.89]**	**0.0009**	0.91 [0.74; 1.12]	0.3946
CMV IgG D+	**1.62 [0.87;3.01]**	**0.1276**	1.34 [0.63; 2.85]	0.4528		
CMV IgG R+	**1.29 [0.70;2.38]**	**0.4098**	1.19 [0.57; 2.49]	0.6442		
CMV IgG D+/R-	**0.86 [0.40;1.84]**	**0.6831**				
No. of infections (y1)	**1.29 [1.17;1.42]**	**<0.0001**	**1.29 [1.17;1.42]**	**<0.0001**	**1.16 [1.03;1.31]**	**0.0114**
Acute rejection						
Agegroup 2	1.24 [0.79; 1.96]	0.3577	1.28 [0.75; 2.20]	0.3621	1.23 [0.71; 2.15]	0.4613
Agegroup 3	1.17 [0.69; 2.00]	0.5582	0.99 [0.37; 2.66]	0.9810	0.96 [0.39; 2.35]	0.9250
Male gender	1.20 [0.82; 1.76]	0.5582	**2.l11 [1.20;3.70]**	**0.0092**	1.83 [1.05; 3.20]	0.0340
BMI (per 5 kg/m^2^)	1.01 [0.97; 1.05]	0.6167	1.04 [0.83; 1.30]	0.7228		
Diabetes mellitus	0.77 [0.45; 1.30]	0.3276	0.64 [0.33; 1.21]	0.1690		
Donor age (per 10 years)	1.01 [1.00; 1.02]	0.0724	1.10 [0.86; 1.39]	0.4427	**0.52 [0.31;0.88]**	**0.0146**
Male donor	**0.64 [0.44;0.95]**	**0.0247**	**0.48 [0.27;0.83]**	**0.0092**		
Deceased donation	0.97 [0.67; 1.42]	0.8879	0.74 [0.40; 1.35]	0.3276		
High sensitization	1.36 [0.79; 2.34]	0.2615	1.01 [0.50; 2.01]	0.9829		
Thymoglobuline	1.21 [0.79; 1.86]	0.3873	0.94 [0.47; 1.89]	0.8639		
HLA mismatches (no.)	1.12 [0.99; 1.26]	0.0723	1.10 [0.93; 1.30]	0.2681		
AB0i	1.01 [0.41; 2.48]	0.9797	1.02 [0.31; 3.32]	0.9801		
Cold ischemia time (min)	1.00 [1.00; 1.00]	0.9620	1.00 [1.00; 1.00]	0.5842		
Delayed graft function	1.05 [0.70; 1.58]	0.8108	0.98 [0.57; 1.69]	0.9372		
CKD-EPI W2	0.99 [0.98; 1.00]	0.0014	0.94 [0.80; 1.10]	0.4634		
CMV IgG D+	1.06 [0.72; 1.55]	0.7217	0.73 [0.42; 1.29]	0.2811		
CMV IgG R+	1.12 [0.76; 1.65]	0.5608	1.06 [0.58; 1.97]	0.8443		
CMV IgG D+/R-	1.00 [0.63; 1.60]	0.9951	0.72 [0.37; 1.42]	0.3480		
Infection (any)						
Agegroup 2	0.95 [0.75; 1.20]	0.6526	0.79 [0.61; 1.02]	0.0742	0.78 [0.59; 1.01]	0.0635
Agegroup 3	**1.46 [1.12;1.91]**	**0.0053**	1.17 [0.82; 1.67]	0.3950	1.20 [0.82; 1.74]	0.3563
Male gender	1.12 [0.92; 1.37]	0.2732	**1.27 [1.02;1.60]**	**0.0347**	1.33 [1.00; 1.76]	0.0466
BMI (per 5 kg/m^2^)	1.01 [0.99; 1.03]	0.5705	1.08 [0.95; 1.22]	0.2431		
Diabetes mellitus	**1.26 [0.98;1.63]**	**0.0704**	1.21 [0.86; 1.69]	0.2744		
Donor age (per 10 years)	**1.01 [1.00;1.02]**	**0.0198**	1.03 [0.96; 1.11]	0.4540		
Male donor	1.03 [0.85; 1.26]	0.7404	1.00 [0.79; 1.27]	0.9980		
Deceased donation	**1.43 [1.16;1.76]**	**0.0009**	1.40 [1.04; 1.88]	0.2830	1.25 [0.80; 1.98]	0.3301
High sensitization	**1.03 [0.75;1.41]**	**0.0704**	1.12 [0.75; 1.67]	0.5871		
Thymoglobuline	1.11 [0.88; 1.40]	0.3857	1.10 [0.84; 1.45]	0.4900		
HLA mismatches (no.)	1.04 [0.98; 1.11]	0.2191	1.00 [0.93; 1.07]	0.9038		
AB0i	1.12 [0.70; 1.79]	0.6439	1.05 [0.61; 1.82]	0.8504		
Cold ischemia time (min)	**1.00 [1.00;1.00]**	**0.0329**	1.00 [1.00; 1.00]	0.0554	1.00 [1.00; 1.00]	0.6809
Delayed graft function	**1.28 [1.03;1.58]**	**0.0250**	1.20 [0.93; 1.54]	0.1720		
CKD-EPI W2	**0.99 [0.99;1.00]**	**0.0046**	0.95 [0.89; 1.01]	0.1080		
CMV IgG D+	0.97 [0.80; 1.19]	0.7832	0.82 [0.64; 1.06]	0.1287		
CMV IgG R+	1.18 [0.96; 1.44]	0.1139	1.15 [0.89; 1.49]	0.2763		
CMV IgG D+/R-	1.11 [0.87; 1.41]	0.4197	0.99 [0.78; 1.28]	0.9663		

Cox regression was performed with and without inverse probability weighting (IPW) based on propensity scores for the exposure age group (reference: age group 1 = recipient age <40 years). The three-step approach includes unadjusted cox (UA), inverse probability weighted univariate (UA-IPW), and IPW-adjusted multivariable (MA-IPW) models, where age group and variables with p < 0.1 in UA-IPW were included in the MA-IPW model. IPW was derived from a multinomial logistic model using clinical and demographic covariates. Only complete cases were analyzed. In the multivariable model, variables with p < 0.1 in IPW-univariable models and clinically relevant variables were included, along with age group regardless of significance.

Abbreviations: Age group 1 = recipients <40 years, age group 2 = recipients 40–60 years, age group 3 = recipients >60 years, AB0i = AB0-incompatible transplantation, BMI = body Mass index (kg/m^2^), CKD-EPI W2 = estimated glomerular filtration rate at week 2 post-transplant (ml/min/1.73 m^2^, CKD-EPI formula), CMV = cytomegalovirus, D+ = donor positive, R+ = recipient positive, R− = recipient negative, D+/R− = donor positive/recipient negative serostatus, DGF = delayed graft function, HR = hazard ratio, CI = confidence interval, HLA = human leukocyte antigen, IPW = inverse probability weighting, MA-IPW = multivariable IPW-weighted model, UA = unadjusted model, UA-IPW = univariate IPW-weighted model, no. of infections (y1) = number of infections during the first post-transplant year. Thymoglobulin = used for induction therapy. Delayed graft function = Defined as the requirement for dialysis within the first 7 days after kidney transplantation, excluding dialysis performed solely for hyperkalemia. Bold values indicate statistically significant results (p < 0.05).

### BKV

BKV viremia was most common in the 40–60 years age group. In the first year post-transplant, the cumulative incidence ranged from 11.6% in recipients <40 years to 15.1% in those >60 years (Supplementary Table S2). New BKV viremia cases appeared in the >60 years group after the first year. Recipients aged 40–60 and >60 years had higher initial plasma viral loads than younger recipients, while those <40 years had the highest peak viral loads. Older recipients (40–60 and >60 years) also had shorter intervals between successive BKV viremia peaks (Supplementary Figure S1). The highest BKV nephropathy (BKVN) rate was found in the 40–60 years group (19.3%), compared to 5.3% in <40 years and 12.5% in >60 years.

### Herpesviruses

CMV viremia was similar across age groups in the first post-transplant year: 13.6% in recipients <40 years, 14.6% in 40–60 years, and 16.4% in those >60 years. Beyond the first year, the CMV incidence continued to rise in older recipients, reaching 21.5% at 5 years (Supplementary Table S2). Peak viral load was highest in recipients >60 years (103,500 IU/mL), followed by <40 years (66,988 IU/mL) and 40–60 years (53,325 IU/mL). CMV-related organ complications occurred more frequent in recipients >60 years (19.2%) than in recipients between 40 and 60 years (17.4%) and <40 years (4.8%). Reinfection rates were significantly higher in recipients >60 years (46.2%) and 40–60 years (45.7%) than in those <40 years (28.6%, p = 0.03). *De novo* CMV infections occurred more frequently in >60 years (46.7%) than in 40–60 years (39.7%) and <40 years (39.3%). Reactivation rates followed a similar trend: 20.0% in >60 years, 14.8% in 40–60 years, and 11.9% in <40 years.

Non-CMV herpesvirus infections had a cumulative incidence of 6.1% at year one, increasing to 11.8% at 5 years. The overall incidence was 13.8%, with most cases occurring after the first year. VZV infections (all herpes zoster) increased from 2.5% at year one to 7.2% at 5 years. Notably, 90% of VZV infections in recipients >60 years occurred after the first year, with a median onset of 27 months (IQR 16–40), compared to 8 months (IQR 2–15) in those <40 years.

HSV infections had a cumulative incidence of 3.1% at year one, 4.5% at 5 years, and 6.0% overall. Nearly 46% of cases were diagnosed after the first year, particularly in older recipients. 61.5% of these cases presented with pneumonia, while all HSV cases in younger recipients were limited to herpes labialis.

Four cases of post-transplant lymphoproliferative disorder (PTLD) were reported, with a median onset of 266 days (IQR 241–335). Seventy-five percent of these cases occurred in recipients >60 years, including one who required transplant nephrectomy.

### Opportunistic Infections With Organ Involvement

The 5-year cumulative incidence of opportunistic infections with organ involvement increased with age, from 3.4% in recipients <40 years to 12.7% in those >60 years (Table 2). Bacterial infections predominated in the youngest group (57.1%), while fungal infections were most common in the 40–60 years group (43.5%), followed by viral (34.8%) and bacterial (21.7%) infections. In recipients >60 years, fungal and viral infections occurred equally (40% each).

The median onset of infections was late (Md = 420 days, IQR 104–948), particularly in older recipients. Pneumonia was the most frequent manifestation, caused by pathogens including HSV (n = 11), Pneumocystis jirovecii (n = 11), Aspergillus spp. (n = 9), CMV (n = 4), *Mycobacterium tuberculosis* (n = 2), *Legionella* (n = 1), and Rhodococcus (n = 1). CMV colitis occurred in 10 cases. Although one case of disseminated Bartonella infection was identified in a 24-year-old, disseminated infections occurred predominantly in recipients >60 years and included severe disease such as mucormycosis (resulting in death within 26 days post-transplant), nocardiosis with brain abscess, invasive aspergillosis, multifocal CMV disease, and cryptococcal pyelonephritis requiring nephrectomy.

Age >60 years was initially associated with a higher risk of opportunistic infections but lost significance after adjustment (HR = 3.20, p = 0.1275; Figure 2), while high sensitization and high BMI remained significant predictors (Supplementary Table S2).

Aspergillus infections occurred exclusively in recipients >40 years of age, with an incidence of 1.8% in the 40–60 years group and 4.6% in those >60 years. Pneumocystis jirovecii pneumonia (PjP) occurred in all age groups but was least common in recipients <40 years (0.6% vs. 2.6% in older groups). Aspergillus infections appeared significantly earlier in the 40–60 years group (median 62 days, IQR 45–78) than in those >60 years (291 days, IQR 63–866; p = 0.037). The majority of PjP cases (90.9%) occurred after prophylaxis was stopped, with no age-related differences in time course. Intensive induction therapy was administered more frequently in PjP cases (33.3%) than in Aspergillus cases (8.3%). Recipients with Aspergillus infections had a higher infection burden in the first post-transplant year (mean 3.6 episodes) than in recipients with PjP (mean 2.2 episodes). Aspergillus-related mortality was 28.3% in recipients >60 years, while no deaths were observed in the 40–60 years group. Although 33.3% of PjP patients died, none of the deaths were directly attributable to PjP.

### Acute Rejections and Transplant Biopsy Findings

Acute rejection occurred in 22.6% of recipients (95% CI: 19.3–26.3), with no significant differences between age groups (Table 2; Figure 1c). However, the timing of rejection varied by age: the earliest rejections were observed in recipients >60 years (median 37 days, IQR 15–118), followed by 40–60 years (65 days, IQR 16–162), and the latest in recipients <40 years (78 days, IQR 13–414).

Recurrent rejection occurred most frequently in the youngest group (8.2%) compared to 5.4% in those >60 years. Biopsy findings revealed only minor age-related differences. Borderline rejection was the most common finding, occurring in 37.8%–47.3% of cases. Acute tubular necrosis increased with age, peaking at 13.3% in recipients >60 years. T-cell–mediated rejection (12.4%) and BKVN (11.8%) occurred most frequently in the 40–60 years group.

### Graft Failure

The 5-year graft failure rate increased significantly with recipient age, from 5.2% (95% CI, 3.0–8.9) in recipients <40 years to 21.0% (95%-CI, 14.6–33.2) in those >60 years (p 0.0001, Table 2; Figure 1b). The unadjusted analysis showed a higher risk of graft loss in recipients >60 years (Figure 2; Table 3). Graft failure also occurred earlier in older recipients, with a median time to failure of 34 months (IQR 18–56) in the >60 group, compared to 52 months (IQR 31–74) in <40 years.

Kidney transplant removal was more often necessary in older recipients (50.2% in >60 years vs. 18.2% in <40 years, p < 0.0001). Infections and acute or chronic rejection were the main causes of graft failure in all age groups. In recipients <40 years, infections accounted for more than half of graft losses, while infections contributed to 40% of graft failures in those >60 years. These infections included BKVN, parainfectious complications, and other graft-related infections.

After adjustment, recipient age >60 years was no longer a significant predictor of graft failure (HR = 0.67, p = 0.3924; Figure 2; Table 3). Independent predictors included donor age, male donor and the number of infections during the first post-transplant year (Table 3).

### Mortality

Five-year mortality rate rised significantly with increasing age, from 1.8% (<40 years) to 18.4% (>60 years) (p < 0.0001) (Table 2; Figure 1d). The unadjusted analysis showed a strong association between age >60 years and mortality risk (Figure 2; Table 3). Even after multivariable adjustment, it remained a significant predictor of mortality (HR = 1.15, p = 0.07), along with higher BMI, donor age and an increased infection burden in the first year after transplantation (Table 3). Sensitivity analyses confirmed the robustness of the association between older recipient age and mortality, whereas no consistent association was observed for graft failure (Supplementary Table S3). Analyses accounting for the COVID-19 pandemic period did not materially change the results (Supplementary Table S4).

Infections were the most common cause of death at 40% of cases. Pulmonary infections were the most frequent, affecting 55.6% of patients. Fatal cases included COVID-19 pneumonia, pulmonary aspergillosis, and *Legionella* pneumonia. Bloodstream infections and sepsis were the second most common causes of death. Pathogens were detected in approximately one-third of deaths, with Aspergillus spp., HSV, and *E. coli* being most common.

In recipients >60 years, the median time to death was shortest (24 months), compared with 28 months in the 40–60 years group and 33 months in <40 years (p = 0.034).

### Risk Factors for First-Year Infection Burden

The first post-transplant year emerged as a critical period linking early infection burden to long-term outcomes. Higher infection burden during the first post-transplant year was independently associated with recipient age >60 years, CMV D+/R− serostatus, higher donor age, and greater HLA mismatch count (Supplementary Table S5).

## Discussion

Our findings demonstrate that recipient age significantly influences post-transplant outcomes, affecting both infection patterns and long-term patient and graft survival. Consistent with previous reports, unadjusted analyses showed higher rates of bacterial and fungal infections, graft failure, and mortality in older recipients [15, 21, 23, 27–32]. They experienced higher rates of pneumonia, UTIs, invasive opportunistic infections, and infections caused by multidrug-resistant organisms. While unadjusted analyses showed broad age-related differences, only a restricted set of associations persisted after adjustment, indicating that many apparent age effects are mediated through modifiable clinical and immunologic factors.

Consistent with Esnault et al., age independently predicted mortality but not overall opportunistic infections [31]. Older recipients were more prone to invasive fungal infections and more severe viral disease, particularly CMV and HSV indicating a pathogen-specific rather than generalized susceptibility. While the overall incidence of CMV infection did not differ by age, older recipients exhibited higher viral loads, more frequent organ involvement, and a greater proportion of late reactivations. Bacterial infection profiles also differed across age groups: *P. aeruginosa* was markedly more prevalent in older recipients and frequently associated with complicated UTIs and urosepsis. Younger recipients, by contrast, tended to experience recurrent but clinically milder lower UTIs. These findings emphasize the importance of age-adjusted infection profiling to tailor prophylaxis and surveillance strategies individually. In older recipients, extended CMV monitoring beyond standard prophylaxis and early assessment of bacterial resistance patterns may be particularly beneficial. Notably, our recent findings within the DZIF transplant cohort revealed that center-specific prophylaxis and monitoring strategies substantially influence herpes- and polyomavirus infection rates, highlighting additional opportunities for optimizing local protocols.

Infections remain one of the leading challenges in post-transplant care, with 54.6% of recipients experiencing at least one infectious complication within the first year [33]. Our analysis revealed that the first year after transplantation is a crucial period for long-term outcomes. A higher infection burden in the early phase was independently associated with recipient age over 60 years, CMV serostatus D+/R–, older donor age, and greater HLA incompatibility. These factors likely interact by increasing infectious exposure and immune stress in the early post-transplant phase, reducing graft resilience and recovery capacity. Although the infection burden contributed to the risk of mortality, it did not fully explain the excess mortality observed in older patients, suggesting reduced physiological and immunological resilience that limits the ability to compensate for infectious and inflammatory stress.

Consistent with previous findings [33], pneumonia was a leading cause of death in recipients over 60, highlighting the need for targeted respiratory surveillance, age-adapted vaccinations, and regular assessment of the net sate of immunosuppression [34]. Fungal infections should be a central component of the differential diagnosis of respiratory symptoms in older recipients. Closer respiratory monitoring, including vigilant clinical examination, early oxygen saturation checks, and early mycological testing can facilitate earlier detection.

Older recipients experienced fewer but delayed acute rejection episodes, whereas younger recipients had more frequent recurrent and early episodes. Consistent with previous reports [27, 35], infection-related morbidity and graft explantation rates were higher in older recipients despite similar rejection rates, indicating that excessive, rather than insufficient immunosuppression in this group. This likely reflects age-related immune alterations that weaken immune defenses and increase infection susceptibility. [36]. Although older recipients received lower tacrolimus doses, trough levels were comparable to younger recipients. Tailoring immunosuppression through pharmacodynamic monitoring may help to balance infection risk [37].

The strengths of this study include the large cohort, the long follow-up period, and the detailed characterization of infectious complications in relation to graft and patient outcomes.

Our multivariable, IPW-based approach minimized confounding and allowed robust assessment of independent age effects. Limitations arise from the non-centralized infection diagnosis, which may have led to some observer variability but reflects clinical practice. The exclusion of incomplete cases may have introduced bias, and some subgroup analyses were limited in their statistical power due to small sample sizes.

In conclusion, age should not be considered a fixed risk factor, but rather a composite marker for immunologic, infectious, and physiological vulnerability. Importantly, the infection burden within the first post-transplant year emerged as a strong independent predictor of both graft failure and mortality, defining a critical, modifiable window for preventive interventions. Post-transplant care in the elderly should therefore primarily include strict infection control, enhanced CMV monitoring, and the early detection of *Pseudomonas* and fungal infections in the first year. Optimizing immunosuppression and minimizing the use of nephrotoxic medications can further protect graft function. Future multicenter studies integrating immunomonitoring, pharmacodynamic profiling, and long-term infection surveillance are needed to further refine these findings.

## Data Availability

The original contributions presented in the study are included in the article/Supplementary Material, further inquiries can be directed to the corresponding author.

## Group Members of the Transplant Cohort of THE German Center for Infection Research (DZIF Transplant Cohort)

Christine S. Falk, Nele Kanzelmeyer, Anette Melk, Thomas F. Schulz, Susanne Delecluse, Philipp Ehlermann, Uta Merle, Burkhard Tönshoff, Joachim Andrassy, Martin Hildebrandt, Michael Neuenhahn, Tina Ganzemüller, Thomas Iftner, Peter Lang, Berit Lange, Carolina Klett-Tammen, Bärbel Fösel, Lutz Renders, and Thomas Illig.
