# Supplementary material for: Age-Related Risk After Kidney Transplantation: A Comprehensive Analysis of Infection Burden, Graft Outcomes, and Mortality
Source: Transpl Int. 2026 Jan 7;38:15267. doi: 10.3389/ti.2025.15267 (PMC12822487; doi:10.3389/ti.2025.15267)

**Supplementary Material**

# **Table 1S.** Infection definitions

Detailed definitions of infections, categorized by infection type.

| **Category** | **Infection Type** | **Definition** |
| --- | --- | --- |
| Bacterial Infections | Urinary tract infection (UTI) | Defined by characteristic symptoms (fever, dysuria, urgency, or frequency) or elevated laboratory markers, combined with significant bacteriuria (>10⁵ CFU/mL in midstream urine). |
|  | Bloodstream infection (BSI) | Defined by at least one positive blood culture with a recognized pathogen or two separate positive blood cultures with a common skin contaminant. |
|  | Sepsis | Defined by an increase of ≥2 points in the SOFA score, along with clinical manifestations such as fever, tachycardia, hypotension, and altered mental status. Diagnosis was supported by a confirmed pathogen in clinical samples. |
|  | Acute graft pyelonephritis | Diagnosed based on at least one positive finding in microbiological/imaging evidence (positive urine dipstick, urine culture, or ultrasound), laboratory markers (leukocyte count ≥12 × 10⁹ cells/L or CRP ≥80 mg/L, or temperature >37.8°C), and clinical signs (graft/flank pain, chills, dysuria, urgency). |
|  | Pneumonia (PNA) | Diagnosed by clinical symptoms (cough, dyspnea, fever), new lung infiltrates on imaging, and optionally confirmed by sputum cultures or PCR. |
|  | Gastrointestinal infections (GI) | Defined by diarrhea, vomiting, or abdominal pain, with positive stool culture or PCR for pathogens (e.g. Clostridium difficile, Salmonella, Norovirus). |
| Viral Infections | Cytomegalovirus (CMV) | CMV infection was confirmed with a viral load ≥5,000 IU/mL in plasma. For data prior to November 2017, a conversion factor of ×3 was applied for conversion from copies/mL to IU/mL. CMV disease required clinical symptoms and evidence of organ involvement, while CMV syndrome included systemic symptoms (fever, malaise, leukopenia) without organ involvement. |
|  | Epstein-Barr Virus (EBV) | EBV infection was significant at a viral load ≥10,000 IU/mL in plasma, with a conversion factor of ×5 used for prior data. Post-transplant lymphoproliferative disorder (PTLD) was diagnosed based on histopathology showing B-cell proliferation and supported by immunohistochemistry and EBV-encoded RNA in situ hybridization. Imaging and clonality assessment were used for staging and diagnosis. |
|  | Herpes Simplex Virus (HSV) & Varicella-Zoster Virus (VZV) | Diagnosed based on clinical presentation (e.g., vesicular rash) and optionally confirmed by PCR. |
|  | BK Virus (BKV) | BKV was considered significant at viral loads >10,000 copies/mL in plasma or biopsy-confirmed BK virus nephropathy (BKVN). |
|  | Respiratory Viral Infections (RVI) | Diagnosed by PCR of respiratory swabs or sputum for influenza, RSV, and other respiratory viruses, in combination with corresponding clinical symptoms. |
| Fungal Infections | Pneumocystis jirovecii Pneumonia (PJP) | Diagnosed by PCR or immunofluorescence staining of BAL fluid, supported by radiological and clinical findings. |
|  | Aspergillosis | Defined by clinical symptoms, radiological imaging (e.g., CT scan), and positive Galactomannan or Aspergillus antigen in BAL/serum. |
|  | Candidemia | Diagnosed by at least one positive blood culture for Candida spp. in the presence of systemic infection signs. Colonization without systemic symptoms was not classified as infection. |
|  | Mucorales | Considered in cases with clinical and radiological evidence of invasive fungal infection, supported by positive culture or PCR for Mucorales spp. |
| Infections of unclear etiology |  | Cases where no definitive pathogen or infection site could be identified were classified as infections of unclear etiology. Diagnosis was based on strong clinical suspicion, laboratory markers (e.g., CRP, PCT), and the patient's response to antimicrobial therapy. |
| Infections were diagnosed and documented by the treating physicians.  Abbreviations:  CFU, colony-forming units; CRP, C-reactive protein; BAL, bronchoalveolar lavage; PCR, polymerase chain reaction; CMV, cytomegalovirus; EBV, Epstein–Barr virus; HSV, herpes simplex virus; VZV, varicella-zoster virus; BKV, BK virus; RSV, respiratory syncytial virus; CT, computed tomography; PJP, Pneumocystis jirovecii pneumonia; PCT, procalcitonin; SOFA, Sequential Organ Failure Assessment; IU, international units. | | |

**Table 2S.** Risk analyses for clinical outcomes using a three-step IPW-adjusted model

|  | **1) Univariate HR (95% CI)** | **p-value** | **2) Univariate IPW HR (95% CI)** | **p-value** | **3) Multivariate IPW HR (95% CI)** | **p-value** |
| --- | --- | --- | --- | --- | --- | --- |
| **Bacterial infection** | | | | | | |
| Agegroup 2 | 0.90 [0-68;1.21] | 0.4967 | 0.82 [0.59;1.13] | 0.2267 | 0.82 [0.59;1.13] | 0.2267 |
| Agegroup 3 | **1.81 [1.22;2.47]** | **0.0002** | **1.77 [1.19;2.65]** | **0.0052** | **1.77 [1.19;2.65]** | **0.0051** |
| Male gender | 0.85 [0.67;1.07] | 0.1730 | 0.86 [0.63;1.17] | 0.3359 |  |  |
| BMI (per 5 kg/m^2^) | 1.00 [0.98;1.03] | 0.8216 | 1.05 [0.90;1.22] | 0.5565 |  |  |
| Diabetes mellitus | 1.28 [0.95;1.72] | 0.1062 | 1.19 [0.82;1.74] | 0.3605 |  |  |
| Donor age (per 10y) | **1.01 [1.00;1.02]** | **0.0272** | 0.95 [0.86;1.05] | 0.3213 |  |  |
| Male donor | 1.10 [1.00;1.02] | 0.4656 | 1.03 [0.75;1.41] | 0.8617 |  |  |
| Deceased donation | **1.46 [1.13;1.88]** | **0.0035** | **1.32 [0.87;1.98]** | **0.1885** |  |  |
| High sensitization | 1.00 [0.68;1.47] | 0.9976 | 1.05 [0.65;1.71] | 0.8320 |  |  |
| Thymoglobuline | 0.98 [0.74;1.30] | 0.8649 | 1.13 [0.80;1.60] | 0.4806 |  |  |
| HLA mismatches | 1.07 [0.99;1.16] | 0.0678 | 0.95 [0.86;1.04] | 0.2401 |  |  |
| AB0i | 1.18 [0.69;2.02] | 0.5418 | 1.07 [0.56;2.06] | 0.8327 |  |  |
| Cold ischemia time (min) | 1.00 [1.00;1.00] | 0.0894 | 1.00 [1.00;1.00] | 0.4268 |  |  |
| Delayed graft function | 1.11 [0.86;1.44] | 0.4199 | 0.92 [0.65;1.28] | 0.6038 |  |  |
| CKD-EPI W2 | **0.99 [0.98;0.99]** | **0.0070** | 0.98 [0.90;1.06] | 0.5880 |  |  |
| CMV IgG D+ | 1.03 [0.81;1.31] | 0.7920 | 0.90 [0.65;1.25] | 0.5313 |  |  |
| CMV IgG R+ | 1.37 [0.87;1.47] | 0.3426 | 1.25 [0.88;1.79] | 0.2136 |  |  |
| CMV IgG D+/R- | 0.89 [0.66;1.21] | 0.4683 | 0.89 [0.60;1.30] | 0.5375 |  |  |
| **Viral infection** | | | | | | |
| Agegroup 2 | 0.96 [0.71;1.28] | 0.7608 | 0.83 [0.60;1.15] | 0.2580 | 0.77 [0.56;1.07] | 0.1167 |
| Agegroup 3 | 1.06 [0.75;1.49] | 0.7569 | 0.73 [0.38;1.39] | 0.3352 | 0.70 [0.46;1.36] | 0.2913 |
| Male gender | 1.28 [0.98;1.66] | 0.0657 | **1.73 [1.11;2.69]** | **0.0152** | **1.60 [1.09;1.31]** | **0.0174** |
| BMI (per 5 kg/m^2^) | 1.01 [0.98;1.03] | 0.5109 | 1.05 [0.89;1.34] | 0.5661 |  |  |
| Diabetes mellitus | 1.08 [0.78;1.49] | 0.6475 | 1.06 [0.70;1.60] | 0.7864 |  |  |
| Donor age (per 10y) | **1.01 [1.00;1.02[** | **0.0027** | **1.19 [1.06;1.35]** | **0.0041** | **1.18 [1.06;1.31]** | **0.0030** |
| Male donor | 0.86 [0.67;1.10] | 0.2248 | 0.97 [0.65;1.45] | 0.8771 |  |  |
| Deceased donation | 1.18 [0.91;1.53] | 0.2170 | 1.17 [0.72;1.91] | 0.5246 |  |  |
| High sensitization | 1.01 [0.74;1.65] | 0.6264 | 1.25 [0.82;1.90] | 0.2959 |  |  |
| Thymoglobuline | 1.05 [0.78;1.41] | 0.7329 | 0.81 [0.49;1.34] | 0.9392 |  |  |
| HLA mismatches | 1.05 [0.97;1.14] | 0.2282 | 1.08 [0.94;1.25] | 0.2558 |  |  |
| AB0i | 0.96 [0.53;1.76] | 0.8967 | 0.97 [0.47;2.01] | 0.9392 |  |  |
| Cold ischemia time (min) | 1.00 [1.00;1.00] | 0.8949 | 1.00 [1.00;1.00] | 0.8911 |  |  |
| Delayed graft function | 1.19 [0.91;1.56] | 0.2037 | 1.29 [0.91;1.83] | 0.1581 |  |  |
| CKD-EPI W2 | 1.00 [0.99;1.00] | 0.1512 | 0.94 [0.84;1.05] | 0.2777 |  |  |
| CMV IgG D+ | 1.00 [0.78;1.29] | 0.9998 | 0.84 [0.56;1.25] | 0.3859 |  |  |
| CMV IgG R+ | 1.09 [0.84;1.40] | 0.5184 | 1.14 [0.73;1.78] | 0.5761 |  |  |
| CMV IgG D+/R- | **1.46 [1.09;1.96]** | **0.1130** | 1.05 [0.56;1.99] | 0.8732 |  |  |
| **Fungal infection** | | | | | | |
| Agegroup 2 | 5.48 [0.70;42.82] | 0.1048 | 3.35 [0.43;25.92] | 0.2464 | 3.67 [0.51;26.64] | 0.1987 |
| Agegroup 3 | **13.80 [1.80;106.27]** | **0.0117** | 6.25 [0.61;64.46] | 0.1240 | 6.91 [0.83;57.81] | 0.0743 |
| Male gender | 0.77 [0.34;1.75] | 0.5289 | 2.01 [0.57;7.14] | 0.2805 |  |  |
| BMI (per 5 kg/m^2^) | 1.07 [0.98;1.15] | 0.1176 | 1.84 [0.53;6.41] | 0.3405 | **1.50 [1.07;2.10]** | **0.0179** |
| Diabetes mellitus | **2.87 [1.22;6.78[** | **0.0160** | 1.29 [1.00;1.67] | 0.0513 |  |  |
| Donor age (per 10y) | **1.04 [1.01;1.07]** | **0.0127** | 0.94 [0.64;1.37] | 0.7337 |  |  |
| Male donor | 1.22 [0.54;2.76] | 0.6366 | 2.19 [0.62;7.74] | 0.2253 |  |  |
| Deceased donation | **3.58 [1.06;12.04]** | **0.0395** | **3.35 [0.81;13.80]** | **0.0948** | 3.82 [0.64;22.81] | 0.1421 |
| High sensitization | 2.69 [1.00;7.25] | 0.0501 | **9.07 [2.47;33.36]** | **0.0009** | **9.86 [2.97;31.49]** | **<0.001** |
| Thymoglobuline | 1.65 [0.62;4.40] | 0.3160 | **2.87 [0.73;11.32]** | **0.1328** |  |  |
| HLA mismatches | 1.09 [0.81;1.46] | 0.5758 | **0.82 [0.48;1.38]** | **0.4480** |  |  |
| AB0i | 0.00 [0.00;0.00] | - | 0.00 [0.00;0.00] | - |  |  |
| Cold ischemia time (min) | 1.00 [1.00;1.00] | 0.1012 | **1.00 [1.00;1.00]** | **0.0210** | 1.00 [1.00;1.00] | 0.4419 |
| Delayed graft function | 1.84 [0.80;4.26] | 0.1523 | 0.80 [0.23;2.75] | 0.7277 |  |  |
| CKD-EPI W2 | 0.98 [0.96;1.00] | 0.0460 | 0.85 [0.68;1.06] | 0.1516 |  |  |
| CMV IgG D+ | 1.65 [0.62;4.41] | 0.3148 | 0.61 [0.16;2.24] | 0.4521 |  |  |
| CMV IgG R+ | 1.45 [0.55;3.87] | 0.4550 | 2.35 [0.70;7.97] | 0.1688 |  |  |
| CMV IgG D+/R- | 1.14 [0.38;3.47] | 0.8151 | 0.72 [0.19;2.78] | 0.6320 |  |  |
| **Urinary tract infection** | | | | | | |
| Agegroup 2 | 1.31 [0.86;2.00] | 0.2144 | 1.17 [0.71;1.95] | 0.5371 | 1.17 [0.71;1.95] | 0.5371 |
| Agegroup 3 | **2.06 [1.31;3.23]** | **0.0016** | 1.66 [0.73;3.74[ | 0.2239 | 1.66 [0.73;3.74] | 0.2239 |
| Male gender | 0.77 [0.56;1.05] | 0.1024 | 1.19 [0.70;2.03] | 0.5139 |  |  |
| BMI (per 5 kg/m^2^) | 1.01 [0.98;1.05] | 0.4544 | 1.08 [0.87;1.33] | 0.5064 |  |  |
| Diabetes mellitus | 1.22 [0.82;1.83] | 0.3330 | 1.11 [0.65;1.89] | 0.7072 |  |  |
| Donor age (per 10y) | 1.01 [1.00;1.02] | 0.1498 | 1.06 [0.90;1.24] | 0.4844 |  |  |
| Male donor | 1.01 [0.74;1.39] | 0.9493 | 1.00 [0.59;1.71] | 0.9900 |  |  |
| Deceased donation | **1.80 [1.25;2.60]** | **0.0016** | 1.35 [0.63;2.90] | 0.4422 |  |  |
| High sensitization | 1.05 [0.63;1.76] | 0.8590 | 1.30 [0.57;2.97] | 0.5360 |  |  |
| Thymoglobuline | 0.79 [0.52;1.20] | 0.2657 | 0.74 [0.38;1.42] | 0.3638 |  |  |
| HLA mismatches | 1.05 [0.94;1.16] | 0.3977 | 0.95 [0.79;1.15] | 0.6130 |  |  |
| AB0i | 0.64 [0.26;1.56] | 0.3250 | 0.76 [0.27;2.18] | 0.6123 |  |  |
| Cold ischemia time (min) | **1.00 [1.00;1.00]** | **0.0258** | 1.00 [1.00;1.00] | 0.5658 |  |  |
| Delayed graft function | 1.36 [0.97;1.90] | 0.0770 | 1.14 [0.70;1.85] | 0.6034 |  |  |
| CKD-EPI W2 | 0.99 [0.99;1.00] | 0.0719 | 0.98 [0.83;1.15] | 0.7918 |  |  |
| CMV IgG D+ | 0.92 [0.66;1.28] | 0.6383 | 0.70 [0.41;1.19] | 0.1863 |  |  |
| CMV IgG R+ | 1.27 [0.90;1.78] | 0.1716 | 1.18 [0.63;2.20] | 0.6009 |  |  |
| CMV IgG D+/R- | 0.98 [0.65;1.48] | 0.9080 | 0.73 [0.38;1.40] | 0.3462 |  |  |
| **Pneumonia** |  |  |  |  |  |  |
| Agegroup 2 | 1.08 [0.62;1.90] | 0.7793 | 1.00 [0.53;1.88] | 0.9881 | 0.85 [0.45;1.60] | 0.6072 |
| Agegroup 3 | **2.62 [1.49;4.62]** | **0.0009** | 1.66 [0.60;4.56] | 0.3251 | 1.84 [0.45;4.60] | 0.1909 |
| Male gender | 1.90 [1.17;3.07] | 0.0088 | **2.89 [1.46;5.73]** | **0.0023** | **2.40 [1.22;4.70]** | **0.0108** |
| BMI (per 5 kg/m^2^) | 1.04 [0.99;1.08] | 0.0841 | 1.03 [0.83;1.29] | 0.7634 | 1.11 [0.86;1.44] | 0.4174 |
| Diabetes mellitus | 1.13 [0.66;1.93] | 0.6661 | 0.63 [0.33;1.20] | 0.1635 |  |  |
| Donor age | **1.04 [1.02;1.05]** | **<0.0001** | **1.33 [1.06;1.66]** | **0.0121** | **1.37 [1.11;1.70]** | **0.0039** |
| Male donor | 1.04 [0.68;1.57] | 0.8690 | 0.95 [0.46;1.95] | 0.8783 |  |  |
| Deceased donation | **1.76 [1.09;2.82]** | **0.0199** | 0.75 [0.41;1.37] | 0.3463 |  |  |
| High sensitization | 1.26 [0.67;2.36] | 0.4767 | 1.00 [0.50;2.00] | 0.9972 |  |  |
| Thymoglobuline | 1.17 [0.72;1.89] | 0.5360 | 0.94 [0.47;1.88] | 0.8586 |  |  |
| HLA mismatches | 1.17 [1.02;1.34] | 0.0227 | 1.10 [0.93;1.30] | 0.2635 |  |  |
| AB0i | 0.74 [0.24;2.35] | 0.6118 | 1.02 [0.31;3.34] | 0.9724 |  |  |
| Cold ischemia time (min) | **1.00 [1.00;1.00]** | **0.0146** | 1.00 [1.00;1.00] | 0.3916 |  |  |
| Delayed graft function | 1.24 [0.79;1.95] | 0.3490 | 0.97 [0.56;1.68] | 0.9215 |  |  |
| CKD-EPI W2 | **0.98 [0.97;0.99]** | **0.0002** | 0.94 [0.81;1.11] | 0.4777 |  |  |
| CMV IgG D+ | **1.11 [0.72;1.70]** | **0.6374** | 0.53 [0.27;1.03] | 0.0599 | **0.49 [0.26;0.90]** | **0.0223** |
| CMV IgG R+ | **1.27 [0.82;1.96]** | **0.2877** | 0.91 [0.42;1.99] | 0.8141 |  |  |
| CMV IgG D+/R- | **0.92 [0.53;1.58]** | **0.7605** | 0.67 [0.79;1.31] | 0.8898 |  |  |
| **Infections with resistant bacteria** | | | | | | |
| Agegroup 2 | 1.81 [0.67;4.90] | 0.2440 | 1.66 [0.55;5.01] | 0.3719 | 1.56 [0.52;4.70] | 0.4267 |
| Agegroup 3 | **3.73 [1.37;10.19]** | **0.0102** | 2.95 [0.57;15.19] | 0.1953 | 3.09 [0.62;15.43] | 0.1683 |
| Male gender | 0.64 [0.34;1.21] | 0.1732 | 0.93 [0.29;3.01] | 0.9058 |  |  |
| BMI | 1.03 [0.97;1.10] | 0.3031 | 0.92 [0.63;1.34] | 0.6738 |  |  |
| Diabetes mellitus | 1.65 [0.87;3.49] | 0.1885 | 1.12 [0.39;3.19] | 0.8281 |  |  |
| Donor age | **1.02 [1.00;1.05]** | **0.0436** | **1.25 [1.03;1.51]** | **0.0219** | **1.26 [1.05;1.52]** | **0.0147** |
| Male donor | 1.05 [0.55;2.01] | 0.8777 | 0.60 [0.29;3.01] | 0.9058 |  |  |
| Deceased donation | 1.58 [0.77;3.26] | 0.2163 | 0.49 [0.13;1.76] | 0.2734 |  |  |
| High sensitization | 0.50 [0.12;2.06] | 0.3337 | 0.51 [0.10;2.66] | 0.4251 |  |  |
| Thymoglobuline | 0.69 [0.29;1.67] | 0.4104 | 0.39 [0.12;1.27] | 0.1174 |  |  |
| HLA mismatches | 1.22 [0.99;1.51] | 0.0665 | 0.98 [0.67;1.44] | 0.9328 |  |  |
| AB0i | 1.20 [0.29;5.00] | 0.7995 | 1.28 [0.26;6.42] | 0.7615 |  |  |
| Cold ischemia time (min) | 1.00 [1.00;1.00] | 0.1015 | 1.00 [1.00;1.00] | 0.6510 |  |  |
| Delayed graft function | 0.88 [0.42;1.85] | 0.7292 | 0.43 [0.15;1.22] | 0.1122 |  |  |
| CKD-EPI W2 | 0.99 [0.98;1.00] | 0.1290 | 1.16 [0.86;1.57] | 0.3389 |  |  |
| CMV IgG D+ | 2.04 [0.97;4.26] | 0.0590 | 0.64 [0.19;2.18] | 0.4736 |  |  |
| CMV IgG R+ | **3.02 [1.32;6.91]** | **0.0090** | 1.02 [0.22;4.63] | 0.9789 |  |  |
| CMV IgG D+/R- | 0.66 [0.26;1.70] | 0.3902 | 0.37 [0.11;1.24] | 0.1075 |  |  |
| **Invasvive opportunistic infection** | | | | | | |
| Agegroup 2 | 2.24 [0.75;6.65] | 0.1475 | 1.67 [0.50;5.64] | 0.4057 | 1.90 [0.54;6.67] | 0.3192 |
| Agegroup 3 | **3.63 [1.17;11.26]** | **0.0267** | 2.65 [0.59;11.80] | 0.2024 | 3.20 [0.72;14.31] | 0.1275 |
| Male gender | 1.60 [0.74;3.44] | 0.2308 | 2.18 [0.77;6.20] | 0.1447 |  |  |
| BMI (per 5 kg/m^2^) | 1.05 [0.99;1.13] | 0.1193 | 1.47 [1.00;2.19] | 0.0526 | **1.60 [1.07;2.39]** | **0.0211** |
| Diabetes mellitus | 1.18 [0.49;2.86] | 0.7118 | 1.71 [0,52;5.69] | 0.3794 |  |  |
| Donor age (per 10y) | **1.03 [1.00;1.07]** | **0.0225** | 1.08 [0.72;1.61] | 0.7161 |  |  |
| Male donor | 1.08 [0.54;2.15] | 0.8316 | 1.62 [0.58;4.56] | 0.3591 |  |  |
| Deceased donation | 2.16 [0.93;5.00] | 0.0719 | **3.39 [1.13;10.17]** | **0.0295** | 1.92 [0.49;7.41] | 0.3468 |
| High sensitization | **3.63 [1.69;7.82]** | **0.010** | **6.99 [2.44;20.02]** | **0.0003** | **6.64 [1.90;23.21]** | **0.0031** |
| Thymoglobuline | **2.12 [1.00;4.49]** | **0.0500** | 0.62 [0.31;1.26] | 0.1859 |  |  |
| HLA mismatches | 1.17 [0.93;1.49] | 0.1795 | 0.99 [0.65;1.52] | 0.9720 |  |  |
| AB0i | 0.62 [0.09;4.57] | 0.6430 | 0.48 [0.06;3.71] | 0.4802 |  |  |
| Cold ischemia time (min) | 1.00 [1.00;1.00] | 0.0515 | **1.00 [1.00;1.00]** | **0.0010** | 1.00 [1.00;1.00] | 0.7707 |
| Delayed graft function | 1.08 [0.50;2.32] | 0.8527 | 0.83 [0.32;2.16] | 0.6982 |  |  |
| CKD-EPI W2 | **0.98 [0.96;1.00]** | **0.0220** | 0.83 [0.68;1.02] | 0.0820 | 0.90 [0.76;1.07] | 0.2375 |
| CMV IgG D+ | 1.56 [0.73;3.36] | 0.2524 | 0.58 [0.22;1.56] | 0.2846 |  |  |
| CMV IgG R+ | 1.94 [0.86;4.38] | 0.1109 | 1.99 [0.68;5.83] | 0.2084 |  |  |
| CMV IgG D+/R- | 0.63 [0.22;1.82] | 0.3983 | 0.40 [0.12;1.39] | 0.1501 |  |  |
| Cox regression was performed with and without inverse probability weighting (IPW) based on propensity scores for the exposure age group (reference: age group 1 = recipient age <40 years). The three-step approach includes unadjusted Cox (UA), inverse probability weighted univariate (UA-IPW), and IPW-adjusted multivariable (MA-IPW) models, where age group and variables with p < 0.1 in UA-IPW were included in the MA-IPW model. IPW was derived from a multinomial logistic model using clinical and demographic covariates. Only complete cases were analyzed. In the multivariable model, variables with p < 0.1 in IPW-univariable models were included, along with age group regardless of significance.  Abbreviations: Age group 1 = recipients <40 years, Age group 2 = recipients 40–60 years, Age group 3 = recipients >60 years, AB0i = AB0-incompatible transplantation, BMI = Body Mass Index (kg/m²), CKD-EPI W2 = estimated glomerular filtration rate at week 2 post-transplant (ml/min/1.73 m², CKD-EPI formula), CMV = Cytomegalovirus, D+ = donor positive, R+ = recipient positive, R− = recipient negative, D+/R− = donor positive/recipient negative serostatus, DGF = delayed graft function, HR = hazard ratio, CI = confidence interval, HLA = human leukocyte antigen, IPW = inverse probability weighting, MA-IPW = multivariable IPW-weighted model, UA = unadjusted model, UA-IPW = univariate IPW-weighted model, no. of infections (y1) = number of infections during the first post-transplant year. Thymoglobulin = used for induction therapy. Delayed graft function = Defined as the requirement for dialysis within the first seven days after kidney transplantation, excluding dialysis performed solely for hyperkalemia.  Due to complete separation (no fungal events among AB0-incompatible recipients), AB0 incompatibility was excluded from fungal models. | | | | | | |

# All sensitivity analyses (Tables S3–S6) were conducted within the revised analytical framework, using stabilized and normalized IPW-weighted Cox models

# **Table S3. Sensitivity Analyses of Age Effect: Model-based Sensitivity Analyses for Age Effect (agegroup 3 vs <40) on Death and Graft Loss**

Sensitivity analyses using unweighted and trimmed IPW Cox models confirmed the robustness of the age effect on mortality, while no consistent association was observed for graft failure.

| **Outcome** | **Model** | **Age effect (HR [95% CI])** | **p-value** |
| --- | --- | --- | --- |
| Graft failure | Main (IPW Cox) | 0.78 (0.31–1.96) | 0.6028 |
| Graft failure | M1: Unweighted Cox | 1.23 (0.49–3.04) | 0.6612 |
| Graft failure | M2: Trimmed IPW (2.5–97.5%) | 0.86 (0.34–2.17) | 0.7561 |
| Mortality | Main (IPW Cox) | 7.20 (1.56–33.14) | 0.0113 |
| Mortality | M1: Unweighted Cox | 7.06 (1.58–31.60) | 0.0106 |
| Mortality | M2: Trimmed IPW (2.5–97.5%) | 8.21 (1.83–36.88) | 0.0061 |
| Abbreviations: IPW, inverse probability weighting; HR, hazard ratio; CI, confidence interval. | | | |

**Table S4. COVID-19 Sensitivity Analyses for Age Effect on Death and Graft Failure**

Two sensitivity analyses were performed to assess the potential influence of the COVID-19 pandemic period on outcomes: (i) censoring follow-up at March 1, 2020 (B1), and (ii) including a pandemic indicator variable for follow-up overlapping March 2020–December 2022 (B2). Results are shown for the age group >60 years versus <40 years. Stabilized IPW-weighted Cox models were used as in the main analysis.

| **Outcome: Death** | | | |
| --- | --- | --- | --- |
| **Model** | **Age effect (HR [95% CI])** | **p-value** | **Notes** |
| Main (IPW Cox) | 6.21 (1.34;28.81) | 0.002 |  |
| B1: Censor follow-up @ 2020-03-01 | 586212664.80 (197329181.44–1741482358.85) | 3.45e-16 | Sparse-events instability before pandemic; interpret with caution. |
| B2: Add pandemic indicator | 4.74 (0.97;23.12) | 0.0014 |  |
| **Outcome: Graft failure** | | | |
| Main (IPW Cox) | 0.99 (0.37–2.68) | 0.9916 |  |
| B1: Censor follow-up @ 2020-03-01 | 6.27 (0.82–48.26) | 0.0776 | Wider CI; fewer events before pandemic. |
| B2: Add pandemic indicator | 0.79 (0.28–2.22) | 0.6531 |  |
| Abbreviations: HR, hazard ratio; CI, confidence interval; IPW, inverse probability weighting. B1 shows sparse-events instability before 2020; interpret with caution. | | | |

# **Table S5. Risk factors for first-year infection burden (Negative Binomial Regression)**

Infection burden refers to the number of clinically significant infections within 12 months post-transplant. *p* < 0.05; **p* < 0.01; ***p* < 0.001. Models were fitted using negative binomial regression with overdispersion correction (φ = 1.79). Rate ratios (RRs) >1 indicate a higher infection burden during the first post-transplant year.

**Table S5A. Univariable negative binomial regression of infection burden during the first post-transplant year.**

All models were weighted by the inverse probability of age group (IPW). Displayed are rate ratios (RR) with 95% confidence intervals. Displayed are rate ratios (RR) with 95% confidence intervals.

| **Variable** | **Rate ratio (95% CI)** | **p-value** |
| --- | --- | --- |
| Recipient age 40–60 years | 1.09 [0.86–1.39] | 0.483 |
| Recipient age >60 years | 1.88 [1.44–2.44] | <0.001*** |
| Recipient male | 0.98 [0.80–1.20] | 0.848 |
| Deceased donation | 1.70 [1.37–2.11] | <0.001*** |
| AB0i | 0.93 [0.57–1.50] | 0.752 |
| High sensitization | 1.06 [0.76–1.48] | 0.731 |
| BMI (per 5 kg/m²) | 1.03 [0.93–1.14] | 0.564 |
| Donor age (per 10 years) | 1.18 [1.11–1.26] | <0.001*** |
| Donor male | 0.95 [0.78–1.16] | 0.614 |
| Diabetes mellitus | 1.10 [0.85–1.42] | 0.479 |
| Delayed graft function | 1.31 [1.06–1.62] | 0.014* |
| Cold ischemia time (per min) | 1.00 [1.00–1.00] | <0.001*** |
| GFR week 2 (per 10 mL/min) | 0.92 [0.88–0.95] | <0.001*** |
| HLA mismatches (count) | 1.10 [1.03–1.17] | 0.005** |
| Thymoglobuline induction | 1.00 [0.79–1.26] | 0.986 |
| CMV donor IgG+ | 1.11 [0.91–1.36] | 0.285 |
| CMV recipient IgG+ | 0.99 [0.81–1.20] | 0.891 |
| CMV D+/R− | 1.25 [0.99–1.59] | 0.061 |
| AB0i, AB0 incompatibility; BMI, body mass index; CMV, cytomegalovirus; +/R−, CMV donor positive/recipient negative serostatus; GFR, glomerular filtration rate; HLA, human leukocyte antigen; RR, rate ratio | | |

**Table S5B. Multivariable negative binomial regression of infection burden during the first post-transplant year**

All models were weighted by inverse probability of age group (IPW). Variables with p < 0.10 in univariable analysis were included, and recipient age group was retained in all models. Dispersion parameter φ = 1.79.

| **Variable** | **Rate ratio (95% CI)** | **p-value** |
| --- | --- | --- |
| Recipient age >60 years | 1.45 [1.05–1.99] | 0.023* |
| CMV D+/R− | 1.53 [1.01–2.30] | 0.044* |
| Donor age (per 10 years) | 1.08 [1.00–1.16] | 0.046* |
| HLA mismatches (count) | 1.08 [1.01–1.15] | 0.033* |
| AB0i, AB0 incompatibility; CMV, cytomegalovirus; +/R−, CMV donor positive/recipient negative serostatus; HLA, human leukocyte antigen; RR, rate ratio | | |

**Figure 1S.** BKV viral plasma loads dynamics across age groups.

Peak and initial plasma BK viral loads, days to peak, and time between peaks are shown for each age group. Viral kinetics were compared among patients aged <40 years, 40–60 years, and >60 years. Boxplots indicate median, interquartile range, and outliers.

**
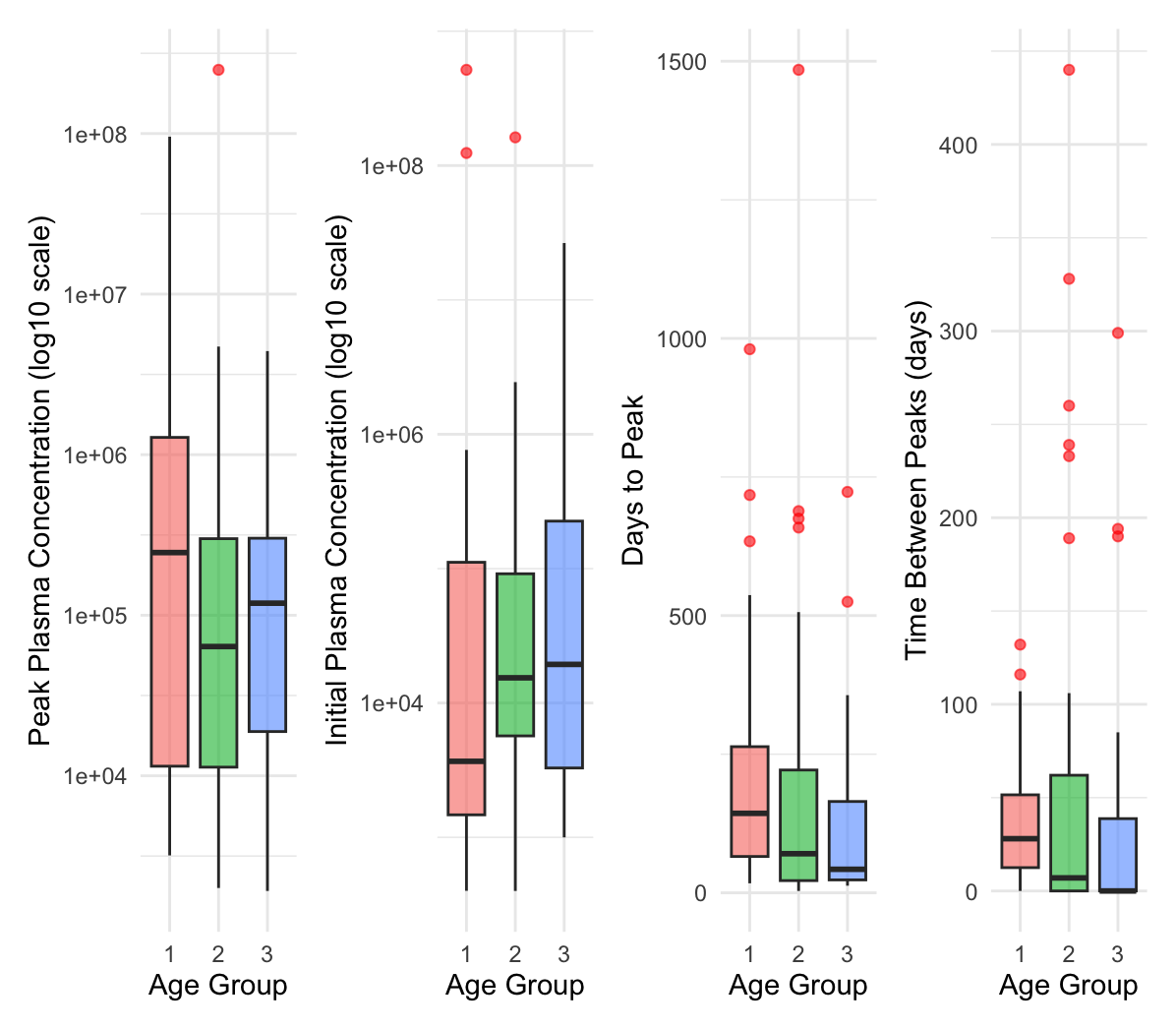
**

**Figure 2S.** Distribution of uropathogens by age group.

Relative proportions of bacterial species isolated from urine cultures across the three age groups (<40 years, 40–60 years, >60 years). The most common pathogens were E. coli, Enterococcus spp., Klebsiella spp., Pseudomonas aeruginosa, and Enterobacter spp., with E. coli predominating in all groups.


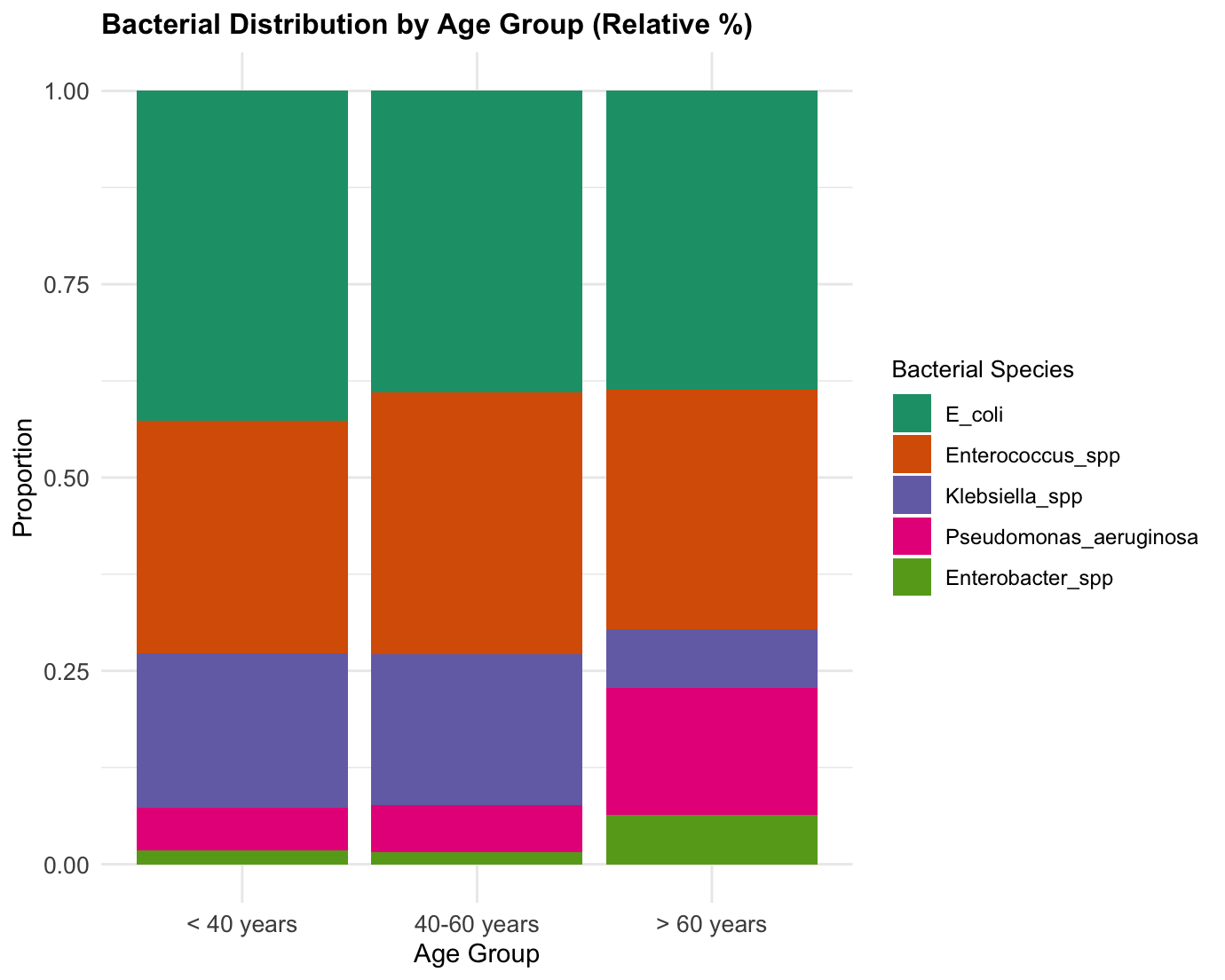

Supplement: Supplementary file 1 [file Supplementaryfile1.docx]
